# Influential journals in health research: a bibliometric study

**DOI:** 10.1186/s12992-016-0186-4

**Published:** 2016-08-22

**Authors:** José M. Merigó, Alicia Núñez

**Affiliations:** Department of Management Control and Information Systems, School of Economics and Business, Universidad de Chile, Diagonal Paraguay 257, Office 2004, Santiago, Chile

**Keywords:** Health, Bibliometrics, Journals, Web of Science

## Abstract

**Background:**

There is a wide range of intellectual work written about health research, which has been shaped by the evolution of diseases. This study aims to identify the leading journals over the last 25 years (1990–2014) according to a wide range of bibliometric indicators.

**Methods:**

The study develops a bibliometric overview of all the journals that are currently indexed in Web of Science (WoS) database in any of the four categories connected to health research. The work classifies health research in nine subfields: Public Health, Environmental and Occupational Health, Health Management and Economics, Health Promotion and Health Behavior, Epidemiology, Health Policy and Services, Medicine, Health Informatics, Engineering and Technology, and Primary Care.

**Results:**

The results indicate a wide dispersion between categories being the American Journal of Epidemiology, Environmental Health Perspectives, American Journal of Public Health, and Social Science & Medicine, the journals that have received the highest number of citations over the last 25 years. According to other indicators such as the *h*-index and the citations per paper, some other journals such as the Annual Review of Public Health and Medical Care, obtain better results which show the wide diversity and profiles of outlets available in the scientific community. The results are grouped and studied according to the nine subfields in order to identify the leading journals in each specific sub discipline of health.

**Conclusions:**

The work identifies the leading journals in health research through a bibliometric approach. The analysis shows a deep overview of the results of health journals. It is worth noting that many journals have entered the WoS database during the last years, in many cases to fill some specific niche that has emerged in the literature, although the most popular ones have been in the database for a long time.

**Electronic supplementary material:**

The online version of this article (doi:10.1186/s12992-016-0186-4) contains supplementary material, which is available to authorized users.

## Background

The scope of public health history is dynamic and has been shaped by the evolution of diseases. In the last decades, health research has moved from the study of sanitary reforms and the control of infectious diseases, to the study of the impact of epidemic and contagious diseases, and to the inclusion of social action initiatives taken in response of epidemic disasters. Thus, the scope of health research has been expanded and broadened from a range of intellectual disciplines including the study of health economics [1], and social and political relations of health [2]. These intellectual contributions have been well documented in a range of professional journals.

The oldest continuously published medical journal was introduced in 1812 by the Massachusetts Medical Society and was called The New England Journal of Medicine and Surgery and the Collateral Branches of Science, which in 1828 became The Boston Medical and Surgical Journal and later The New England Journal of Medicine. From that period of time many other medical associations and journals started to emerge including the Journal of the American Medical Association in 1883, Milbank Quarterly in 1923, Public Health Economics and Medical Care Abstracts (today’s Medical Care Research and Review) in 1944, Inquiry and Medical Care in 1963, Health Service Research in 1966, and Social Science and Medicine in 1967. Healthcare Financial Management and the International Journal of Health Services appeared in the 70’s, and also the Journal of Health Politics, Policy and Law, Health Care Management Review, Hospital and Health Services Administration, the Journal of Ambulatory Care Management and, and Health Care Financing Review. In the 80’s there was an explosion of new journals such as the Journal of Public Health Policy, Health Affairs, Advances in Health Economics and Health Services Research, Health Care Strategic Management, Frontier of Health Services Management, International Journal of Technology Assessment in Health Care, International Journal of Health Planning and Management, Health Care Financing Review, and the International Journal for Quality in Health Care. The first issue of the Journal of Health Services Research and Policy was published in 1996, and in the 2000s the Health Services and Outcomes Research Methodology appeared [3].

For this reason, there is a wide range of intellectual work written about health research and it is time to evaluate its development and main characteristics. Bibliometric analysis can help determine trends and patterns of publications within a research discipline, identifying the focus of research and the national and international strengths and biases [4, 5]. Over the course of the past few decades, several bibliometric analyses have been published regarding diverse topics in the literature of health research, including medicine and international health, where studies have tried to evaluate the contribution of different world regions in research production in infection diseases in terms of both quantity and quality [6], in respiratory research productivity where countries from Western Europe, Canada and Oceania had the best performance after adjustment for population and gross national income per capita [7], in cardiovascular research production where the results showed a promising trend of research productivity for developing world regions with the exception of Africa [8], and also look at the bibliometric profile of tropical medicine and international health [9]. In health informatics studies have included an up-to-date view of the telemedicine and telehealth literature analyzing the changes in content themes [10], assessing the impact of grants in the contribution to scientific knowledge of health information technologies on patient safety and quality of care outcomes [11], quantifying the number of publications on electronic health records and their changes over time [12], and studying the contribution of individual countries to leading journals in medical informatics [13]. Among occupational health, articles have helped to identify the most pertinent journals in the practice of occupational health and to develop search strategies that facilitate finding occupational health intervention studies [14–16]. There are also bibliometric analysis published in health management that investigate the attention and progress of strategic management in the literature [17], in health economics, studies have reported the growth of health economics literature and the change in topics and geographical focus of health economics [18–20]. Bibliometric studies in health service research have looked for trends, gaps and characteristics when comparing among different regions [21, 22], in the same line other studies have assessed the scientific production in health policy [23, 24], in primary health [25, 26], in epidemiology [27, 28], in environmental health [29–31], in public health and preventive medicine [32, 33], in preventive medicine, occupational and environmental medicine, epidemiology, and public health [34], and also public in health research in specific countries or regions [35–42]. This literature has become relevant today perhaps due to the diverse disciplines that are engaged in health research and its importance in order to monitor and disseminate the scientific achievements from the research work.

In this current study we aim to expand the scope of analysis of health research even more, identifying and quantifying the scholarly publications in the field and subfield approaches of public health, environmental and occupational health, health management and economics, health promotion and health behavior, epidemiology, health policy and services, medicine, health informatics, engineering and technology and primary care. Our main goal is to produce an overview of the leading journals in health research literature in the world for a time frame of 25 years from 1990 to 2014. There are many aspects that can indicate the value of a journal; in this study we use the number of citations and their impact factor to reflect the quality of the review process and its popularity in the scientific community. Findings from this research may assist researchers to identify top performance journals in a subject area, learn more about the subfields of health research, and identify emerging areas of research to locate areas of potential study.

## Methods

There are different approaches for classifying the bibliographic material. Bibliometrics is one of the most common approaches [43, 44]. In order to represent the results, the analysis uses a wide range of indicators that measure the quality of the publications. Currently, there is no general agreement on the optimal method for measuring research because depending on the perspective considered an indicator may be better than another one. Therefore, the current literature deals with many indicators that have appeared during the last years [45–47]. In this study, the aim is to provide a general picture in order to get a complete overview that can adapt to the interests and perspectives of each specific reader. Thus, several indicators are considered including the total number of publications and citations, the citations per paper, the *h*-index [48] and the number of papers that a journal has among the most cited ones in a specific field. Note that the most cited papers are found in Web of Science (WoS) by ordering the results of a search from the most cited papers to the less cited [49].

The study uses the WoS database in order to search for the information. WoS is usually regarded as the most influential database for scientific research because it includes those journals that are generally recognized with the highest quality. It was originally created by Eugene Garfield with the name of Institute for Scientific Information (ISI). Later, Thomson & Reuters bought the ISI, renaming it ISI Web of Knowledge and later WoS. Note that other databases are available and could also be used in the analysis including Scopus and Google Scholar. Scientific research is divided in 251 categories in WoS. Four of them involve health research: Public, Environmental & Occupational Health, Health Policy & Services, Health Care Sciences & Services, and Primary Health Care. Note that the first one appears both in the Science Citation Index and in the Social Science Citation Index. These four categories encompass 363 journals. Since these four categories are very broad, the study divides health research in nine categories in order to get a deeper picture of the leading journals in each subfield. However, it is worth noting that sometimes it was not clear where to classify a journal because its scope may include two or three subfields. In any case, the analysis considers a Global Ranking (GR) where all the journals are ranked together. This allows comparison between subfields when needed. The nine categories are:

- Public Health
- Environmental and Occupational Health
- Health Management and Economics
- Health Promotion and Health Behavior
- Epidemiology
- Health Policy and Services
- Medicine
- Health Informatics, Engineering and Technology
- Primary Care

Although the work considers many indicators, the ranking is carried out according to the total number of citations. The main reason for this is that the citations measure the influence that a journal has in absolute terms. Other alternatives could be considered including the citations per paper and the *h*-index. The main limitation of this indicator is that it does not take into account the number of papers that have generated the number of cites. However, the rest of indicators included in the tables allow the reader to get a comprehensive overview of each journal although the ranking is focused on the number of citations.

The analysis focuses on the last 25 years (1990–2014). The main reason is because this time period is representative of the latest developments of the field. In order to take into account the influence from a classical perspective, the study includes an indicator that shows the number of articles that a journal has among the 200 most cited articles published before 200. This allows the reader to see those journals that were very influential before and see if they are still leading the field or not. Note that the search process carried out in WoS has been developed between March and August 2015. The types of documents considered are: articles, reviews, notes and letters.

## Results

This section presents the main bibliometric results found in WoS for 363 health research journals from 1990 to 2014. The results are ordered by category and each table contains the 20 or 10 most cited journals of each category (see the Additional file 1 for the full list of journals).

### Public health

Public health refers to the activities to prevent diseases, promote health, and prolong life for the whole population [50]. Therefore, the main objective of public health is assuring conditions for the people in order to be healthy. It is at the core of health research and consequently there are many journals devoted to increase knowledge and raise awareness on this topic. Table 1 shows the most influential journals among this category.

**Table 1:** Most influential journals in Public Health Research

R	Journal name	TC	TP	H	TC/TP	Year	Volume	IF	IF5	T50	T200	T200*	GR	First year
1	American Journal of Public Health	302196	9203	198	32,84	1991	81	4,229	4,997	15	9	1	3	1911
2	Social Science & Medicine	281663	9656	163	29,17	1990	31	2,558	3,568	17	14	0	4	1967
3	Bulletin of The World Health Organization	80812	2597	110	31,12	1990	68	5,112	6,372	8	7	14	22	1948
4	BMC Public Health	60880	7434	64	8,19	2001	1	2,321	2,781	0	0	0	31	2001
5	Public Health Nutrition	46882	2988	84	15,69	1999	2	2,483	2,798	1	1	0	41	1998
6	Drug Safety	46562	2005	87	23,22	1990	5	2,620	3,424	0	0	0	42	1986
7	Annual Review of Public Health	39592	595	104	66,54	1991	12	6,627	7,984	4	4	1	51	1980
8	Public Health Reports	34617	2064	67	13,29	1990	105	1,644	1,791	1	1	2	61	2001
9	Australian and New Zealand Journal of Public Health	22531	2219	54	10,15	1996	20	1,897	1,835	0	0	0	82	1977
10	Canadian Journal of Public Health-Revue Canadienne de Sante Publique	20835	2515	44	8,28	1990	81	1,094	1,325	1	1	0	88	1997
11	European Journal of Public Health	20399	1851	51	11,02	1997	7	2,459	2,743	0	0	0	91	1991
12	Public Health	20393	2488	48	8,2	1990	104	1,475	1,514	0	0	0	92	1888
13	Revista de Saude Publica	18366	2810	41	6,54	1990	24	1,219	1,587	0	0	0	97	1967
14	Scandinavian Journal of Public Health	17440	1568	50	11,12	1999	27	3,125	2,570	0	0	0	103	1973
15	Journal of American College Health	15664	1011	57	15,49	1994	43	1,397	2,223	2	1	0	109	1978
16	Journal of Safety Research	13917	1070	47	13,01	1990	21	1,303	1,940	0	0	0	116	1982
17	Public Health Nursing	11167	1373	37	8,13	1990	7	0,886	1,131	0	0	0	137	1984
18	Maternal and Child Health Journal	9992	1434	36	6,97	2004	8	2,015	2,318	0	0	0	143	1997
19	Salud Publica de Mexico	8805	2008	30	4,38	1993	35	1,034	1,221	0	0	0	154	1997
20	Ethnicity & Disease	7952	1285	32	6,19	2004	14	0,921	1,233	0	0	0	160	2003

*Abbreviations*: *TC* Total citations, *TP* Total papers, *H* Hirsch index, *TC/TP* Average citations per paper, *IF* Impact factor, *IF5* 5-year impact factor, *T50* Number of papers among the 50 most cited of the category, T200 and T200* Number of papers among the 200 most cited in all fields of health between 1990–2014 and before 1990, *GR* Global ranking considering all the journals

The journal that has received more citations and the higher h-index in this field is the American Journal of Public Health with 302,196 citations and an *h*-index of 198; followed by Social Science & Medicine with 281,663 citations and an *h*-index of 163. These two journals dominate the number of citations in the field with more than 50 % of all the cites. Despite these elevated numbers, the highest average citation rate per paper, impact factor, and 5-year impact factor is for the Annual Review of Public Health.

Analyzing the T50 and T200 ranking results, Social Science & Medicine is in the top positions with 17 papers or 34 % of the papers in the T50 ranking of the category, followed by the American Journal of Public Health with 30 % of the papers in the T50. The same journals have 9 and 14 papers respectively in the T200 ranking. Note that all the journals of the category have an *h*-index bigger than 20, reflecting its influence and productivity.

### Environmental and occupational health

Environmental health studies the impact of our surroundings in health, while occupational health studies all aspects of health and safety in the workplace. This category includes journals regarding the understanding of the impact and control of environmental and occupational hazards on human health and society. Table 2 presents the results for this category.

**Table 2:** Most influential journals in Environmental and Occupational Health Research

R	Journal name	TC	TP	H	TC/TP	Year	Volume	IF	IF5	T50	T200	T200*	GR	First year
1	Environmental Health Perspectives	311509	8066	192	38,62	1990	89	7,029	7,607	40	22	3	2	1972
2	Occupational and Environmental Medicine	71955	3211	98	22,41	1994	51	3,234	3,466	0	0	1	26	1944
3	American Journal of Industrial Medicine	62605	3745	76	16,72	1990	18	1,590	1,899	1	1	1	29	1980
4	Environmental Research	62104	2695	91	23,04	1990	53	3,951	4,033	1	0	0	30	1967
5	Accident Analysis and Prevention	60584	3760	83	16,11	1990	22	2,571	3,096	0	0	0	32	1969
6	Journal of Occupational and Environmental Medicine	57259	4235	80	13,52	1990	32	1,797	2,09	0	0	1	34	1959
7	Radiation Protection Dosimetry	53396	9063	55	5,89	1990	30	0,861	0,981	0	0	0	36	1981
8	Scandinavian Journal of Work Environment & Health	44939	1853	83	24,25	1990	16	3,095	3,869	3	1	0	45	1975
9	International Archives of Occupational and Environmental Health	37358	2381	66	15,69	1990	62	2,198	2,199	1	1	0	56	1930
10	Aviation Space and Environmental Medicine	34588	4265	51	8,11	1990	61	0,782	0,998	0	0	0	62	1930
11	Annals of Occupational Hygiene	22748	1844	52	12,34	1990	34	2,068	2,148	0	0	0	79	1958
12	Indoor Air	20871	948	62	22,02	1994	4	4,904	-	0	0	0	87	1991
13	Health & Place	19584	1384	58	14,15	1995	1	2,435	3,003	0	0	0	95	1995
14	Occupational Medicine-Oxford	19400	2080	53	9,33	1992	42	1,472	1,682	1	0	0	96	1948
15	Journal of Toxicology and Environmental Health-Part A-Current Issues	17092	1670	44	10,23	1998	53	1,834	1,868	0	0	0	104	1975
16	International Journal of Hygiene and Environmental Health	15445	1080	51	14,3	2000	203	3,276	3,331	0	0	0	110	2000
17	Toxicology and Industrial Health	15004	1429	49	10,5	1990	6	1,710	1,591	3	0	0	115	1985
18	Journal of Environmental Science and Health Part B-Pesticides Food Contaminants and Agricultural Wastes	13210	1901	34	6,95	1990	25	1,234	1,129	0	0	0	122	1976
19	Environmental Geochemistry and Health	12095	1093	42	11,07	1990	12	2,573	2,534	0	0	0	128	1979
20	Industrial Health	10652	1444	35	7,38	1990	28	1,045	1,132	0	0	0	140	1963

*Abbreviations*: *TC* Total citations, *TP* Total papers, *H* Hirsch index, *TC/TP* Average citations per paper, *IF* Impact factor, *IF5* 5-year impact factor, *T50* Number of papers among the 50 most cited of the category, T200 and T200* Number of papers among the 200 most cited in all fields of health between 1990–2014 and before 1990, *GR* Global ranking considering all the journals

The journal Environmental Health Perspectives has the highest results in all the measures, with 311,509 citations that are far from any other journal in the category, an *h*-index of 192, and an impact factor of 7.029. It has also 80 % of the papers in the T50 ranking, and 22 papers in the T200 ranking of the most cited papers in all fields of health.

### Health management and economics

This category includes journals related to the economics and management of health and healthcare (see Table 3).

**Table 3:** Most influential journals in Health Management and Economics Research

R	Journal name	TC	TP	H	TC/TP	Year	Volume	IF	IF5	T50	T200	T200*	GR	First year
1	Medical Care	162015	3890	145	41,65	1990	28	2,941	3,714	31	16	10	9	1963
2	Risk Analysis	50164	2716	85	18,47	1990	10	1,974	2,546	5	2	1	38	1981
3	Journal of Health Economics	39818	1400	85	28,44	1990	9	2,254	3,159	5	3	1	49	1982
4	Pharmacoeconomics	37078	2163	69	17,04	1993	4	3,338	3,509	2	0	0	57	1992
5	Medical Decision Making	34695	1511	80	22,96	1990	10	2,698	3,083	5	1	1	59	1981
6	Health Economics	32797	1643	72	19,96	1994	3	2,137	2,570	2	0	0	65	1992
7	BMC Health Services Research	25343	3474	49	7,3	2001	1	1,659	2,188	0	0	0	72	2001
8	American Journal of Managed Care	24099	2483	57	9,71	1997	3	2,166	2,688	0	0	0	76	1995
9	Inquiry-The Journal of Health Care Organization Provision and Financing	9704	711	43	13,65	1990	97	0,564	0,793	0	0	0	146	1964
10	Health Care Management Review	6392	702	32	9,11	1994	19	1,642	2,071	0	0	0	179	1976
11	American Journal of Medical Quality	5264	698	30	7,5	1999	14	1,776	1,821	0	0	0	189	1986
12	Journal of Managed Care Pharmacy	4630	616	31	7,52	2006	12	2,682	2,858	0	0	0	198	1995
13	Journal of Public Health Management and Practice	3661	865	21	4,23	2005	11	0,84	1,198	0	0	0	211	1995
14	International Journal of Health Planning and Management	3470	512	26	6,78	1994	9	0,971	1,029	0	0	0	213	1985
15	Economics & Human Biology	3326	348	29	9,56	2006	4	2,461	2,878	0	0	0	217	2003
16	Journal of Healthcare Management	2919	514	24	5,68	1998	43	0,96	0,977	0	0	0	224	2007
17	European Journal of Health Economics	2403	495	19	4,85	2007	8	1,913	1,865	0	0	0	230	2000
18	Australian Health Review	2098	669	17	3,14	2007	31	1,000	1,127	0	0	0	236	1978
19	Human Resources for Health	2048	371	20	5,52	2008	6	1,922	2,392	0	0	0	237	2003
20	International Journal for Equity in Health	1761	458	16	3,84	2008	7	1,589	1,925	0	0	0	247	2002

*Abbreviations*: *TC* Total citations, *TP* Total papers, *H* Hirsch index, *TC/TP* Average citations per paper, *IF* Impact factor, *IF5* 5-year impact factor, *T50* Number of papers among the 50 most cited of the category, T200 and T200* Number of papers among the 200 most cited in all fields of health between 1990–2014 and before 1990, *GR* Global ranking considering all the journals

Medical Care is the journal positioned number one in this category according to the number of citations, *h*-index, average citations per paper, and 5-year impact factor. It has 62 % of the papers among the 50 most cited in the category, and 16 out of the 200 most cited in all fields of health. However, the highest impact factor in the category is for Pharmaeconomics, positioned fourth according to the total number of citations.

### Health promotion and health behavior

Health promotion and health behavior refers to the role of behavioral and social influences to address current and emerging public health problems. Table 4 shows the most influential journals in this category.

**Table 4:** Most influential journals in Health Promotion and Health Behavior

R	Journal name	TC	TP	H	TC/TP	Year	Volume	IF	IF5	T50	T200	T200*	GR	First year
1	American Journal of Preventive Medicine	115942	3947	133	29,37	1990	6	4,281	5,092	13	5	5	12	1985
2	Preventive Medicine	111066	4186	122	26,53	1990	19	2,932	3,917	4	3	3	14	1972
3	Journal of Adolescent Health	78449	3676	96	21,34	1990	11	2,748	3,753	2	0	0	23	1980
4	Patient Education and Counseling	59813	3611	83	16,56	1990	15	2,598	3,158	2	0	0	33	1978
5	Medical Education	50964	3253	79	15,67	1990	24	3,617	3,963	0	0	0	37	1966
6	Journal of Health and Social Behavior	45820	728	107	62,94	1990	31	2,951	4,457	8	4	4	44	1960
7	Supportive Care in Cancer	39781	3384	62	11,76	1993	1	2,495	2,845	0	0	0	50	1993
8	AIDS Care-Psychological and Socio-Medical Aspects of AIDS/HIV	39017	2795	66	13,96	1992	4	2,194	2,454	1	1	1	53	1989
9	American Journal of Community Psychology	33534	1259	84	26,64	1990	18	1,968	2,888	1	1	0	63	1973
10	Tobacco Control	32078	1641	74	19,55	1998	7	5,150	4,532	1	0	0	66	1992
11	Health Education Research	31136	1594	73	19,53	1991	6	1,944	2,508	0	0	0	67	1986
12	Qualitative Health Research	28115	1864	58	15,08	1995	5	1,441	-	1	1	0	70	1991
13	Psychology & Health	24761	1407	57	17,6	1992	7	2,255	2,107	5	1	1	74	1987
14	Medical Teacher	24402	3370	52	7,24	1990	12	2,045	2,170	1	1	1	75	1979
15	Sociology of Health & Illness	23121	1134	65	20,39	1990	12	2,014	2,62	0	0	0	77	1979
16	AIDS and Behavior	22636	1863	55	12,15	2003	7	3,312	3,977	0	0	0	81	1997
17	American Journal of Health Promotion	21372	1072	63	19,94	1995	9	1,762	2,389	4	2	2	83	1986
18	AIDS Education and Prevention	20901	1123	57	18,61	1992	4	1,505	2,298	0	0	0	86	1989
19	Journal of School Health	20436	1887	56	10,83	1990	60	1,659	2,132	0	0	0	90	1930
20	AIDS Patient Care and STDs	20210	1622	49	12,46	1996	10	3,576	3,255	0	0	0	93	1987

*Abbreviations*: *TC* Total citations, *TP* Total papers, *H* Hirsch index, *TC/TP* Average citations per paper, *IF* Impact factor, *IF5* 5-year impact factor, *T50* Number of papers among the 50 most cited of the category, T200 and T200* Number of papers among the 200 most cited in all fields of health between 1990–2014 and before 1990, *GR* Global ranking considering all the journals

As we can see in Table 4, there are two journals leading the citation ranking: the American Journal of Preventive Medicine and Preventive Medicine with 115,942 and 111,066 citations respectively. Also these two journals have an *h*-index over 100. The Tobacco Control Journal has the highest impact factor of 5150; however, the American Journal of Preventive Medicine has the highest 5-year impact factor. Additionally, the American Journal of Preventive Medicine has 13 studies in the T50 ranking and five in the T200.

### Epidemiology

Epidemiology is a basic science of public health. It studies the distributions and determinants of health problems in different groups of people. Epidemiological information is used to plan and evaluate strategies to control or prevent diseases. Table 5 presents the main journals in this category.

**Table 5:** Most influential journals in Epidemiology

R	Journal name	TC	TP	H	TC/TP	Year	Volume	IF	IF5	T50	T200	T200*	GR	First year
1	American Journal of Epidemiology	357243	8162	211	43,77	1990	131	4,975	6,067	11	8	49	1	1921
2	Cancer Epidemiology Biomarkers & Prevention	207614	6073	147	34,19	1991	1	4,324	4,647	2	1	0	5	1991
3	Journal of Clinical Epidemiology	168450	4502	167	37,42	1990	43	5,478	5,898	17	16	2	7	1955
4	Statistics in Medicine	148692	6505	140	22,86	1990	9	2,037	2,828	0	0	6	10	1982
5	International Journal of Epidemiology	140691	4335	134	32,45	1990	19	9,197	8,000	3	0	3	11	1972
6	Journal of Epidemiology and Community Health	99343	3939	117	25,22	1990	44	3,294	3,667	2	2	3	15	1947
7	Epidemiology	93837	3124	126	30,04	1991	2	6,178	6,894	1	1	0	16	1990
8	Infection Control and Hospital Epidemiology	86969	4588	100	18,96	1990	11	3,938	4,423	5	3	0	18	1980
9	Cancer Causes & Control	83834	3003	112	27,92	1990	1	2,961	3,434	1	1	0	21	1990
10	Epidemiology and Infection	73121	4117	85	17,76	1990	104	2,491	2,671	0	0	0	25	1901
11	European Journal of Epidemiology	46372	2896	71	16,01	1990	6	5,147	4,245	1	1	0	43	1985
12	Annals of Epidemiology	43089	2002	83	21,52	1996	6	2,145	2,895	0	0	0	48	1990
13	Genetic Epidemiology	38300	2040	77	18,77	1990	7	2,951	3,489	1	1	1	54	1984
14	Epidemiologic Reviews	34630	456	93	75,94	1990	12	7,333	12,344	4	3	5	60	1979
15	Community Dentistry and Oral Epidemiology	33089	1726	66	19,17	1990	18	1,944	2,491	0	0	0	64	1973
16	Neuroepidemiology	24773	1314	62	18,85	1990	9	2,476	2,863	0	0	0	73	1982
17	Paediatric and Perinatal Epidemiology	21249	1228	59	17,3	1994	8	2,811	2,796	0	0	0	84	1987
18	Journal of Urban Health-Bulletin of the New York Academy of Medicine	18231	1274	54	14,31	1998	75	1,943	2,517	0	0	0	99	1925
19	Statistical Methods in Medical Research	11801	490	44	24,08	1999	8	2,957	3,155	2	2	0	131	1992
20	BMC Medical Research Methodology	8936	979	39	9,13	2007	7	2,168	3,024	0	0	0	153	2001

*Abbreviations*: *TC* Total citations, *TP* Total papers, *H* Hirsch index, *TC/TP* Average citations per paper, *IF* Impact factor, *IF5* 5-year impact factor, *T50* Number of papers among the 50 most cited of the category, T200 and T200* Number of papers among the 200 most cited in all fields of health between 1990–2014 and before 1990, *GR* Global ranking considering all the journals

The American Journal of Epidemiology is in the first place of this category, and also among all the categories according to the global ranking (GR). It has also the highest *h*-index of all categories, reflecting his influence and impact. It is followed by the Cancer Epidemiology Biomarkers & Prevention and the Journal of Clinical Epidemiology that occupy positions 5 and 7 in the GR. The highest impact factor and 5-year impact factor is for the Epidemiology journal with values of 6178 and 6894 respectively. Regarding the T50 ranking, the Journal of Clinical Epidemiology has 46 % of the most cited papers in this category and the American Journal of Epidemiology has 22 %. In the T200 ranking, this category has 39 out of the 200 most cited journals in all the fields, eight are from the American Journal of Epidemiology and 16 of the Journal of Clinical Epidemiology.

### Health policy and services

This category includes journals from multidisciplinary fields of scientific investigation that study among others the rising demand for health services, how to improve their quality and efficiency, and rigorous policy analysis that ultimately impact our health and well-being (see Table 6).

**Table 6:** Most influential journals in Health Policy and Services

R	Journal name	TC	TP	H	TC/TP	Year	Volume	IF	IF5	T50	T200	T200*	GR	First year
1	Health Affairs	85858	5495	106	15,62	1990	9	4,321	4,402	11	1	1	19	1981
2	Quality of Life Research	65856	2697	102	24,42	1993	2	2,864	3,270	15	3	3	28	1992
3	Health Services Research	44904	1997	82	22,49	1990	24	2,491	2,772	4	0	0	46	1967
4	Health Policy	29530	1646	53	11,16	1990	14	1,725	1,923	1	1	1	68	1980
5	Milbank Quarterly	22965	605	69	37,96	1990	68	5,391	6,513	8	3	3	78	1923
6	Health Policy and Planning	20646	1306	56	15,81	1991	6	3,442	3,703	1	0	0	89	1986
7	International Journal for Quality in Health Care	18340	1299	54	14,12	1995	7	1,584	2,296	1	0	0	98	1989
8	Value in Health	18129	1403	53	12,92	2002	5	2,891	3,174	3	0	0	100	1998
9	Community Mental Health Journal	15045	1391	49	10,82	1990	26	1,146	1,422	0	0	0	113	1965
10	Medical Care Research and Review	13328	636	55	20,96	1995	52	2,600	3,638	2	0	0	119	1944
11	Hastings Center Report	11744	2179	47	5,39	1990	20	1,080	1,131	0	0	0	132	1971
12	International Journal of Health Services	11664	1102	46	10,58	1990	20	0,988	1,236	2	0	0	133	1971
13	Health and Quality of Life Outcomes	11595	1199	44	9,67	2006	4	2,099	3,152	0	0	0	135	2003
14	Journal of Aging and Health	11313	857	45	13,2	1995	7	1,832	2,057	0	0	0	136	1989
15	Journal of Health Politics Policy and Law	10243	1023	41	10,01	1990	15	0,962	1,169	0	0	0	141	1976
16	Journal of Health Care for the Poor and Underserved	9865	1466	35	6,73	1995	6	0,902	1,394	0	0	0	144	1990
17	Journal of Community Health	9787	1247	39	7,85	1994	19	1,573	1,765	0	0	0	145	1975
18	Psychology Public Policy and Law	9354	508	45	18,41	1995	1	1,723	2,697	2	0	0	149	1995
19	Journal of Rural Health	8759	943	37	9,29	1996	12	1,771	1,844	0	0	0	156	1985
20	Gesundheitswesen	5518	1610	23	3,43	2000	62	0,624	0,621	0	0	0	187	1946

*Abbreviations*: *TC* Total citations, *TP* Total papers, *H* Hirsch index, *TC/TP* Average citations per paper, *IF* Impact factor, *IF5* 5-year impact factor, *T50* Number of papers among the 50 most cited of the category, T200 and T200* Number of papers among the 200 most cited in all fields of health between 1990–2014 and before 1990, *GR* Global ranking considering all the journals

Even though Health Affairs is the most cited journal, the Milbank Quarterly has the highest TC/TP ratio, impact factor, and 5-year impact factor, being the most influential in the category. It is also worthiest to notice that Quality of Life Research has 15 out of the 50 most influential papers in the category, followed by Health Affairs with 11 and Milbank Quarterly with eight. Quality of Life Research and Milbank Quarterly have three papers each in the T200 ranking.

### Medicine

Medicine is one of the world’s oldest categories in the arena of health research, allowing scientists and physicians to communicate their advances. The results of this category are displayed in Table 7.

**Table 7:** Most influential journals in Medicine

R	Journal name	TC	TP	H	TC/TP	Year	Volume	IF	IF5	T50	T200	T200*	GR	First year
1	American Journal of Tropical Medicine and Hygiene	178865	7809	120	22,9	1990	42	2,736	2,947	9	1	1	6	1921
2	Journal of General Internal Medicine	114351	4688	120	24,39	1990	5	3,423	3,744	14	3	3	13	1986
3	Academic Medicine	92662	7000	98	13,24	1990	65	3,468	3,654	12	1	1	17	1926
4	Transactions of the Royal Society of Tropical Medicine and Hygiene	84422	4933	83	17,11	1990	84	1,931	2,453	1	0	0	20	1908
5	Psychiatric Services	74932	4731	91	15,84	1995	46	1,987	2,807	6	0	0	24	1950
6	Journal of Pain and Symptom Management	71194	3586	95	19,85	1991	6	2,737	3,240	2	0	0	27	1986
7	Tropical Medicine & International Health	56435	3196	74	17,66	1996	1	2,302	2,953	0	0	0	35	1996
8	Nicotine & Tobacco Research	28496	1975	61	14,43	2003	5	2,805	3,125	2	0	0	69	1999
9	Palliative Medicine	25715	1711	63	15,03	1995	9	2,845	3,565	0	0	0	71	1987
10	Journal of Womens Health	20943	1932	49	10,84	1997	6	1,896	1,989	1	0	0	85	1992
11	Journal of Manipulative and Physiological Therapeutics	18094	2429	45	7,45	1990	13	1,248	1,471	1	1	1	101	1978
12	Annals of Human Biology	15402	1414	44	10,89	1990	17	1,148	1,515	0	0	0	111	1974
13	Journal of Evaluation in Clinical Practice	13335	1785	44	7,47	1999	5	1,580	1,534	0	0	0	118	1995
14	Vector-Borne and Zoonotic Diseases	13124	1248	41	10,52	2003	3	2,531	2,635	0	0	0	124	2001
15	Journal of Public Health Dentistry	11983	1012	46	11,84	1990	50	1,644	1,653	0	0	0	130	1941
16	Journal of Palliative Medicine	11142	1586	39	7,03	2005	8	2,063	2,446	0	0	0	138	1987
17	European Journal of Cancer Care	9674	1056	40	9,16	1999	8	1,762	1,813	0	0	0	147	1992
18	Journal of Medical Screening	9179	675	41	13,6	1998	5	2,722	2,234	0	0	0	150	1994
19	Implementation Science	8777	883	39	9,94	2006	1	3,470	4,098	1	0	0	155	2006
20	Medical Anthropology Quarterly	8041	608	40	13,23	1990	4	0,607	1,117	0	0	0	159	1987

*Abbreviations*: *TC* Total citations, *TP* Total papers, *H* Hirsch index, *TC/TP* Average citations per paper, *IF* Impact factor, *IF5* 5-year impact factor, *T50* Number of papers among the 50 most cited of the category, T200 and T200* Number of papers among the 200 most cited in all fields of health between 1990–2014 and before 1990, *GR* Global ranking considering all the journals

American Journal of Tropical Medicine and Hygiene is the most influential according to its total number of citations of 178,865. However, the h-index is the same for the American Journal of Tropical Medicine and Hygiene and the Journal of General Internal Medicine, but the TC/TP ratio and impact factor is higher for the latter. The highest impact factor and 5-year impact factor is for the Implementation Science journal. In the T50 ranking, Academic Medicine appears with 12 papers, the American Journal of Tropical Medicine and Hygiene with 9, and the Journal of General Internal Medicine has 14 in this ranking and three in the T200 ranking.

### Health informatics, engineering and technology

This category involves the research and integration of resources, devices, and methodologies to optimize the use of information to provide a better healthcare. The Top ten journals in this category are shown in Table 8.

**Table 8:** Most influential journals in Health Informatics, Engineering and Technology

R	Journal name	TC	TP	H	TC/TP	Year	Volume	IF	IF5	T50	T200	T200*	GR	First year
1	Journal of the American Medical Informatics Association	47258	3139	94	15,06	2000	7	3,932	4,182	26	0	0	40	1994
2	Health Physics	39417	4799	62	8,21	1990	58	0,774	1,105	4	1	1	52	1958
3	International Journal of Medical informatics	22736	1696	55	13,41	1997	44	2,716	-	5	0	0	80	1970
4	Methods of information in Medicine	17886	1704	47	10,5	1990	24	1,083	1,448	3	0	0	102	1962
5	Journal of Telemedicine and Telecare	16334	1980	45	8,25	1998	4	1,736	1,661	0	0	0	106	1995
6	Journal of Medical Internet Research	15683	1271	53	12,34	1999	1	4,669	5,724	4	0	0	108	1999
7	International Journal of Technology Assessment in Health Care	15141	1346	45	11,25	1995	11	1,556	1,565	3	0	0	112	1985
8	Health Technology Assessment	13181	650	56	20,28	2004	8	5,116	5,404	5	1	0	123	1997
9	Journal of Medical Systems	5709	1188	26	4,81	1992	16	1,372	1,482	0	0	0	186	1977
10	International Journal of Health Geographics	4350	485	28	8,97	2007	6	1,967	2,675	0	0	0	202	2002

*Abbreviations*: *TC* Total citations, *TP* Total papers, *H* Hirsch index, *TC/TP* Average citations per paper, *IF* Impact factor, *IF5* 5-year impact factor, *T50* Number of papers among the 50 most cited of the category, T200 and T200* Number of papers among the 200 most cited in all fields of health between 1990–2014 and before 1990, *GR* Global ranking considering all the journals

The Journal of the American Medical Informatics Association is the most influential in this category with the highest number of citations, *h*-index, TC/TP ranking, impact factor, 5-year impact factor. Additionally, it has 52 % of the papers among the 50 most cited of the category, but none in the T200. Health Physics and Health Technology Assessment have one paper each in the T200 ranking.

### Primary care

Primary care is all the regular health services and social services that are available for the population, and its research is at the cornerstone for building a strong healthcare system. Table 9 presents the Top ten journals in this category.

**Table 9:** Most influential journals in Primary Care

R	Journal name	TC	TP	H	TC/TP	Year	Volume	IF	IF5	T50	T200	T200*	GR	First year
1	British Journal of General Practice	47635	5770	74	8,26	1990	40	2,356	2,516	8	0	0	39	1953
2	American Family Physician	43288	5726	62	7,56	1990	41	1,818	2,056	4	0	0	47	1970
3	Journal of Family Practice	37511	3371	78	11,13	1990	30	0,735	0,729	17	1	1	55	1974
4	Family Practice	35533	2322	66	15,3	1990	7	1,842	2,071	6	0	0	58	1984
5	Annals of Family Medicine	16159	666	59	24,26	2004	2	4,570	5,250	9	0	0	107	2003
6	Canadian Family Physician	13319	5046	35	2,64	1990	36	1,403	1,646	0	0	0	120	1955
7	Family Medicine	10804	1546	38	6,99	2000	32	0,851	1,284	3	1	1	139	1981
8	Scandinavian Journal of Primary Health Care	9132	866	35	10,55	1995	13	1,610	1,889	0	0	0	152	1983
9	Journal of the American Board of Family Medicine	7230	898	32	8,05	2006	19	1,848	2,064	2	0	0	168	1988
10	Primary Care	6856	1244	28	5,51	1990	17	0,833	1,084	1	0	0	174	1974

*Abbreviations*: *TC* Total citations, *TP* Total papers, *H* Hirsch index, *TC/TP* Average citations per paper, *IF* Impact factor, *IF5* 5-year impact factor, *T50* Number of papers among the 50 most cited of the category, T200 and T200* Number of papers among the 200 most cited in all fields of health between 1990–2014 and before 1990, *GR* Global ranking considering all the journals

The most cited journal is the British Journal of General Practice, followed by the American Family Physician, the Journal of Family Practice, and Family Practice. The highest *h*-index is for the Journal of Family Practice. While these four papers are the most cited, Annals of Family Medicine is the journal with the highest impact factor, and 5-year impact factor. Among the 50 most cited papers in this category, the Journal of Family Practice has 34 % of them, followed by Annals of Family Medicine with 18 %, the British Journal of General Practice with 16 %, and Family Practice with 12 %. There are two journals in the T200 ranking with one paper each, which are the Journal of Family Practice and Family Medicine.

### Comparison between fields

Finally, let us provide a general picture of the publication and citation structure of each health category. Table 10 presents the results.

**Table 10:** Global results for each category

Category name	NJ	H	TC	TP	TC/TP	T200	T200*
Public Health	61	261	1.237.899	83.365	14,85	38	28
Environmental and Ocupational Health	47	215	1.091.045	73.649	14,81	25	6
Health Management and Economics	29	188	474.540	28.547	16,62	22	13
Health Promotion and Health Behaviour	73	218	1.156.553	78.542	14,73	21	19
Epidemiology	25	325	1.806.202	66.097	27,33	39	69
Health Policy and Services	40	172	484.055	37.911	12,77	8	8
Medicine	51	184	951.188	69.198	13,75	6	6
Health Informatics, Engineering and Technology	18	123	214.135	21.205	10,10	2	1
Primary Health Care	18	113	250.486	34.269	7,31	2	2

Note that the columns of the T200 and the T200* do not sum up to 200 because some of the most cited papers were published in journals that currently are not indexed in WoS for several reasons

*Abbreviations*: *NJ* Number of journals

Epidemiology is the most influential category over the last 25 years with the highest number of citations, citations per paper, *h*-index and the largest number of articles among the 200 most cited before and after 1990. Public Health also obtains very remarkable results being the category with the largest number of articles. However, the category with the highest number of journals is Health Promotion and Health Behavior.

## Discussion and conclusions

This study showed some remarkable viewpoints about health journals currently indexed in WoS database between 1990 and 2014. This analysis shows the results obtained by health journals under a wide range of bibliometric indicators. This is very useful to see the general results from a broader perspective than the Journal Citation Reports of WoS. Particularly, this is very useful for PhD students and newcomers in the field in order to get a general orientation of the leading journals in health research.

The results provide a general picture of the current position of the leading journals in this field for the nine categories analyzed, which can be rank in terms of total papers as follows: (1) Public Health, (2) Health Promotion and Health Behavior, (3) Medicine, (4) Environmental and Occupational Health, (5) Epidemiology, (6) Health Policy and Services, (7) Health Management and Economics, (8) Health Informatics, Engineering and Technology, and (9) Primary Care. The results indicate that Epidemiology is the most influential category although Public Health is the largest one. Focusing on journals, the American Journal of Epidemiology, Environmental Health Perspectives, American Journal of Public Health, and Social Science & Medicine, have the highest number of citations which shows the largest influence in absolute terms. However, some other journals achieve better results when looking to other indicators. For example, according to the citations per paper ratio, the leading journals are Epidemiologic Reviews, Annual Review of Public Health, and Journal of Health and Social Behavior. This indicates that the journals considered in the study have different profiles with a wide variety of objectives. Almost all the leading journals are published in English although some journals may also publish in some other popular European languages including German, Spanish, French and Italian. Note that many journals have entered the WoS database during the last years although the most popular ones have been in the database for a long time.

In conclusion, by bibliometric methodology, the findings and suggestions of this study can help scientific researchers understand the performance and trends of health research globally. With the help of these findings researchers can make informed decisions of their research directions, in terms of identifying top journals of the discipline, and choose exchange platforms for their research. This study also helps to learn more about the subfields of health research, and identify emerging areas of research today such as public health, health promotion and health behavior and epidemiology, and also how most areas of health research have started to decline in number of citations from 2006.

From a general perspective, health research is a very broad area that includes a wide range of topics. This article classifies the material according to some of the most significant topics. However, it is worth noting that more specific topics could also be considered because each journal considers specific topics that do not depend on the topics followed by other journals. Moreover, the study assigns each journal to one category but sometimes the journal could also be considered in another category. For solving this issue, the last column of each table (GR) considers the global ranking that each journal obtains when merging all the journals in the same list and ordering according to the number of citations. This allows comparison between journals from different categories. But note that each category or group of journals may have a different profile with a different volume of publications and citations. Therefore, it is not easy to compare journals with different topics and categories. Finally, recall that WoS has several limitations when classifying bibliographic material that should also be considered in this article [49]. Additionally, many other issues may affect research in this field including open access and electronic journals. Note that only those included in WoS are considered in the analysis.

## Additional file

Additional file 1:
**Table S1.** Public Health. **Table S2.** Environmental and Occupational Health. **Table S3.** Health Management and Economics. **Table S4.** Health Promotion and Health Behavior. **Table S5.** Epidemiology. **Table S6.** Health Policy and Services. **Table S7.** Medicine. **Table S8.** Health Informatics, Engineering and Technology (e-health). **Table S9.** Primary Health Care. (DOCX 153 kb)

## Abbreviations

GR: Global Ranking
WoS: Web of Science
