# Supplementary material for: Influential journals in health research: a bibliometric study
Source: Global Health. 2016 Aug 22;12(1):46. doi: 10.1186/s12992-016-0186-4 (PMC4994291; doi:10.1186/s12992-016-0186-4)
Supplement: Additional file 1: — Table S1. Public Health. Table S2. Environmental and Occupational Health. Table S3. Health Management and Economics. Table S4. Health Promotion and Health Behavior. Table S5. Epidemiology. Table S6. Health Policy and Services. Table S7. Medicine. Table S8. Health Informatics, Engineering and Technology (e-health). Table S9. Primary Health Care. (DOCX 153 kb) [file 12992_2016_186_MOESM1_ESM.docx]

**Additional file 1**

Table S1. Public Health

| R | Journal Name | TC | TP | H | TC/TP | Year | Volume | IF | IF5 | T50 | T200 | T200* | GR | First Year |
| --- | --- | --- | --- | --- | --- | --- | --- | --- | --- | --- | --- | --- | --- | --- |
| 1 | American Journal of Public Health | 302196 | 9203 | 198 | 32,84 | 1991 | 81 | 4,229 | 4,997 | 15 | 9 | 1 | 3 | 1911 |
| 2 | Social Science & Medicine | 281663 | 9656 | 163 | 29,17 | 1990 | 31 | 2,558 | 3,568 | 17 | 14 | 0 | 4 | 1967 |
| 3 | Bulletin of The World Health Organization | 80812 | 2597 | 110 | 31,12 | 1990 | 68 | 5,112 | 6,372 | 8 | 7 | 14 | 22 | 1948 |
| 4 | BMC Public Health | 60880 | 7434 | 64 | 8,19 | 2001 | 1 | 2,321 | 2,781 | 0 | 0 | 0 | 31 | 2001 |
| 5 | Public Health Nutrition | 46882 | 2988 | 84 | 15,69 | 1999 | 2 | 2,483 | 2,798 | 1 | 1 | 0 | 41 | 1998 |
| 6 | Drug Safety | 46562 | 2005 | 87 | 23,22 | 1990 | 5 | 2,620 | 3,424 | 0 | 0 | 0 | 42 | 1986 |
| 7 | Annual Review of Public Health | 39592 | 595 | 104 | 66,54 | 1991 | 12 | 6,627 | 7,984 | 4 | 4 | 1 | 51 | 1980 |
| 8 | Public Health Reports | 34617 | 2064 | 67 | 13,29 | 1990 | 105 | 1,644 | 1,791 | 1 | 1 | 2 | 61 | 2001 |
| 9 | Australian and New Zealand Journal of Public Health | 22531 | 2219 | 54 | 10,15 | 1996 | 20 | 1,897 | 1,835 | 0 | 0 | 0 | 82 | 1977 |
| 10 | Canadian Journal of Public Health-Revue Canadienne de Sante Publique | 20835 | 2515 | 44 | 8,28 | 1990 | 81 | 1,094 | 1,325 | 1 | 1 | 0 | 88 | 1997 |
| 11 | European Journal of Public Health | 20399 | 1851 | 51 | 11,02 | 1997 | 7 | 2,459 | 2,743 | 0 | 0 | 0 | 91 | 1991 |
| 12 | Public Health | 20393 | 2488 | 48 | 8,2 | 1990 | 104 | 1,475 | 1,514 | 0 | 0 | 0 | 92 | 1888 |
| 13 | Revista de Saude Publica | 18366 | 2810 | 41 | 6,54 | 1990 | 24 | 1,219 | 1,587 | 0 | 0 | 0 | 97 | 1967 |
| 14 | Scandinavian Journal of Public Health | 17440 | 1568 | 50 | 11,12 | 1999 | 27 | 3,125 | 2,570 | 0 | 0 | 0 | 103 | 1973 |
| 15 | Journal of American College Health | 15664 | 1011 | 57 | 15,49 | 1994 | 43 | 1,397 | 2,223 | 2 | 1 | 0 | 109 | 1978 |
| 16 | Journal of Safety Research | 13917 | 1070 | 47 | 13,01 | 1990 | 21 | 1,303 | 1,940 | 0 | 0 | 0 | 116 | 1982 |
| 17 | Public Health Nursing | 11167 | 1373 | 37 | 8,13 | 1990 | 7 | 0,886 | 1,131 | 0 | 0 | 0 | 137 | 1984 |
| 18 | Maternal and Child Health Journal | 9992 | 1434 | 36 | 6,97 | 2004 | 8 | 2,015 | 2,318 | 0 | 0 | 0 | 143 | 1997 |
| 19 | Salud Publica De Mexico | 8805 | 2008 | 30 | 4,38 | 1993 | 35 | 1,034 | 1,221 | 0 | 0 | 0 | 154 | 1997 |
| 20 | Ethnicity & Disease | 7952 | 1285 | 32 | 6,19 | 2004 | 14 | 0,921 | 1,233 | 0 | 0 | 0 | 160 | 2003 |
| 21 | Cadernos de Saude Publica | 7803 | 2158 | 28 | 3,62 | 2010 | 26 | 0,888 | 1,321 | 0 | 0 | 0 | 163 | 1985 |
| 22 | Journal of Public Health | 7460 | 853 | 33 | 8,75 | 2004 | 26 | 2,296 | 2,312 | 0 | 0 | 10 | 166 | 1979 |
| 23 | Journal of Health Population and Nutrition | 6574 | 867 | 30 | 7,58 | 2000 | 18 | 1,388 | 1,541 | 0 | 0 | 0 | 177 | 2000 |
| 24 | Revista Panamericana De Salud Publica-Pan American Journal of Public Health | 6053 | 1325 | 26 | 4,57 | 2004 | 16 | 0,723 | 0,998 | 0 | 0 | 0 | 182 | 1997 |
| 25 | Bundesgesundheitsblatt-Gesundheitsforschung -Gesundheitsschutz | 4995 | 1417 | 31 | 5,53 | 2007 | 50 | 1,006 | 0,798 | 0 | 0 | 0 | 192 |  |
| 26 | Ethnicity & Health | 4862 | 430 | 30 | 11,31 | 2000 | 5 | 1,276 | 1,983 | 0 | 0 | 0 | 194 | 1996 |
| 27 | International Journal of Public Health | 4065 | 615 | 26 | 6,61 | 2007 | 52 | 1,966 | 2,605 | 0 | 0 | 0 | 207 | 1956 |
| 28 | Health | 3711 | 400 | 29 | 9,28 | 2001 | 5 | 1,324 | 1,648 | 0 | 0 | 0 | 210 | 2009 |
| 29 | Ciencia & Saude Coletiva | 3351 | 2210 | 16 | 1,52 | 2008 | 13 | 0,572 | 0,684 | 0 | 0 | 0 | 215 | 1996 |
| 30 | Journal of Immigrant and Minority Health | 3042 | 848 | 19 | 3,59 | 2008 | 10 | 1,264 | 1,454 | 0 | 0 | 0 | 222 | 1999 |
| 31 | Mmwr-Morbidity and Mortality Weekly Report | 2333 | 454 | 22 | 5,14 | 2013 | 62 | N/A | N/A | 0 | 0 | 0 | 231 | 1982 |
| 32 | Gaceta Sanitaria | 2155 | 832 | 15 | 2,59 | 2007 | 21 | 1,250 | 1,294 | 0 | 0 | 0 | 234 | 1987 |
| 33 | Rural and Remote Health | 2021 | 730 | 15 | 2,77 | 2008 | 8 | 0,868 | 1,106 | 0 | 0 | 0 | 238 | 2001 |
| 34 | Biosecurity and Bioterrorism-Biodefense Strategy Practice and Science | 1992 | 343 | 21 | 5,81 | 2004 | 2 | 1,618 | 1,532 | 0 | 0 | 0 | 239 | 2003 |
| 35 | Globalization and Health | 1897 | 310 | 20 | 6,12 | 2005 | 1 | 1,833 | 2,267 | 0 | 0 | 0 | 243 | 2005 |
| 36 | Public Health Genomics | 1895 | 251 | 22 | 7,55 | 2009 | 12 | 2,462 | 2,389 | 0 | 0 | 0 | 244 | 1998 |
| 37 | Who Technical Report Series | 1749 | 410 | 15 | 4,27 | 1990 | - | 0,732 | N/A | 1 | 0 | 0 | 249 | 1947 |
| 38 | Asia-Pacific Journal of Public Health | 1707 | 516 | 14 | 3,31 | 2006 | 18 | 1,111 | 1,312 | 0 | 0 | 0 | 251 | 1987 |
| 39 | Iranian Journal of Public Health | 1655 | 1198 | 13 | 1,38 | 2006 | 35 | 0,576 | 0,679 | 0 | 0 | 0 | 252 | 1972 |
| 40 | Disaster Medicine and Public Health Preparedness | 1586 | 468 | 17 | 3,39 | 2007 | 1 | 1,142 | 1,256 | 0 | 0 | 0 | 253 | 2007 |
| 41 | Global Public Health | 1353 | 454 | 14 | 2,98 | 2009 | 4 | 1,205 | N/A | 0 | 0 | 0 | 257 | 2006 |
| 42 | Population Health Metrics | 1288 | 171 | 18 | 7,53 | 2010 | 8 | 2,537 | N/A | 0 | 0 | 0 | 260 | 2003 |
| 43 | Global Health Action | 1198 | 542 | 13 | 2,21 | 2008 | 1 | 1,646 | 2,302 | 0 | 0 | 0 | 265 | 2008 |
| 44 | Revista Espanola de Salud Publica | 1140 | 502 | 13 | 2,27 | 2006 | 80 | 0,600 | 0,804 | 0 | 0 | 0 | 268 | 1926 |
| 45 | BMC International Health and Human Rights | 954 | 239 | 14 | 3,99 | 2009 | 9 | 1,096 | N/A | 0 | 0 | 0 | 278 | 2001 |
| 46 | International Health | 702 | 243 | 12 | 2,89 | 2009 | 1 | 1,129 | 1,183 | 0 | 0 | 0 | 291 | 2009 |
| 47 | Public Health Ethics | 646 | 182 | 12 | 3,55 | 2008 | 1 | 1,269 | 1,023 | 0 | 0 | 0 | 293 | 2008 |
| 48 | Critical Public Health | 458 | 167 | 11 | 2,74 | 2010 | 20 | 1,282 | N/A | 0 | 0 | 0 | 313 | 1990 |
| 49 | Saude e Sociedade | 457 | 659 | 7 | 0,69 | 2008 | 17 | 0,174 | 0,272 | 0 | 0 | 0 | 314 | 1992 |
| 50 | Lancet Global Health | 417 | 165 | 11 | 2,53 | 2013 | 1 | N/A | N/A | 0 | 0 | 0 | 315 | 2013 |
| 51 | International Journal of Qualitative Studies on Health and Well-Being | 302 | 238 | 6 | 1,27 | 2009 | 4 | 0,296 | N/A | 0 | 0 | 0 | 329 | 2006 |
| 52 | Ethiopian Journal of Health Development | 245 | 164 | 6 | 1,49 | 2009 | 23 | 0,226 | N/A | 0 | 0 | 0 | 334 | 1987 |
| 53 | Central European Journal of Public Health | 239 | 196 | 7 | 1,22 | 2011 | 19 | 0,798 | N/A | 0 | 0 | 0 | 336 | 1993 |
| 54 | Health Reports | 233 | 148 | 7 | 1,57 | 2009 | 20 | 3,314 | N/A | 0 | 0 | 0 | 337 | 1990 |
| 55 | Sante Publique | 190 | 444 | 4 | 0,43 | 2009 | 21 | 0,276 | 0,206 | 0 | 0 | 0 | 342 | 1989 |
| 56 | Mmwr Recommendations and Reports | 147 | 16 | 6 | 9,19 | 2013 | 62 | N/A | N/A | 0 | 0 | 0 | 346 | 1952 |
| 57 | Mmwr Surveillance Summaries | 141 | 22 | 8 | 6,41 | 2013 | 62 | N/A | N/A | 0 | 0 | 0 | 347 | 1983 |
| 58 | Zdravstveno Varstvo | 120 | 177 | 5 | 0,68 | 2009 | 48 | 0,732 | N/A | 0 | 0 | 0 | 351 | 1961 |
| 59 | Salud Colectiva | 118 | 167 | 4 | 0,71 | 2007 | 3 | 0,442 | 0,346 | 0 | 0 | 0 | 352 | 2005 |
| 60 | Eastern Mediterranean Health Journal | 117 | 268 | 4 | 0,44 | 2013 | 19 | N/A | N/A | 0 | 0 | 0 | 353 | 1995 |
| 61 | Annals of Global Health | 9 | 41 | 1 | 0,22 | 2014 | 80 | N/A | N/A | 0 | 0 | 0 | 362 | 1934 |

Table S2. Environmental and Occupational Health

| R | Journal Name | TC | TP | H | TC/TP | Year | Volume | IF | IF5 | T50 | T200 | T200* | GR | First Year |
| --- | --- | --- | --- | --- | --- | --- | --- | --- | --- | --- | --- | --- | --- | --- |
| 1 | Environmental Health Perspectives | 311509 | 8066 | 192 | 38,62 | 1990 | 89 | 7,029 | 7,607 | 40 | 22 | 3 | 2 | 1972 |
| 2 | Occupational and Environmental Medicine | 71955 | 3211 | 98 | 22,41 | 1994 | 51 | 3,234 | 3,466 | 0 | 0 | 1 | 26 | 1944 |
| 3 | American Journal of Industrial Medicine | 62605 | 3745 | 76 | 16,72 | 1990 | 18 | 1,590 | 1,899 | 1 | 1 | 1 | 29 | 1980 |
| 4 | Environmental Research | 62104 | 2695 | 91 | 23,04 | 1990 | 53 | 3,951 | 4,033 | 1 | 0 | 0 | 30 | 1967 |
| 5 | Accident Analysis and Prevention | 60584 | 3760 | 83 | 16,11 | 1990 | 22 | 2,571 | 3,096 | 0 | 0 | 0 | 32 | 1969 |
| 6 | Journal of Occupational and Environmental Medicine | 57259 | 4235 | 80 | 13,52 | 1990 | 32 | 1,797 | 2,09 | 0 | 0 | 1 | 34 | 1959 |
| 7 | Radiation Protection Dosimetry | 53396 | 9063 | 55 | 5,89 | 1990 | 30 | 0,861 | 0,981 | 0 | 0 | 0 | 36 | 1981 |
| 8 | Scandinavian Journal of Work Environment & Health | 44939 | 1853 | 83 | 24,25 | 1990 | 16 | 3,095 | 3,869 | 3 | 1 | 0 | 45 | 1975 |
| 9 | International Archives of Occupational and Environmental Health | 37358 | 2381 | 66 | 15,69 | 1990 | 62 | 2,198 | 2,199 | 1 | 1 | 0 | 56 | 1930 |
| 10 | Aviation Space and Environmental Medicine | 34588 | 4265 | 51 | 8,11 | 1990 | 61 | 0,782 | 0,998 | 0 | 0 | 0 | 62 | 1930 |
| 11 | Annals of Occupational Hygiene | 22748 | 1844 | 52 | 12,34 | 1990 | 34 | 2,068 | 2,148 | 0 | 0 | 0 | 79 | 1958 |
| 12 | Indoor Air | 20871 | 948 | 62 | 22,02 | 1994 | 4 | 4,904 | - | 0 | 0 | 0 | 87 | 1991 |
| 13 | Health & Place | 19584 | 1384 | 58 | 14,15 | 1995 | 1 | 2,435 | 3,003 | 0 | 0 | 0 | 95 | 1995 |
| 14 | Occupational Medicine-Oxford | 19400 | 2080 | 53 | 9,33 | 1992 | 42 | 1,472 | 1,682 | 1 | 0 | 0 | 96 | 1948 |
| 15 | Journal of Toxicology and Environmental Health-Part A-Current Issues | 17092 | 1670 | 44 | 10,23 | 1998 | 53 | 1,834 | 1,868 | 0 | 0 | 0 | 104 | 1975 |
| 16 | International Journal of Hygiene and Environmental Health | 15445 | 1080 | 51 | 14,3 | 2000 | 203 | 3,276 | 33,331 | 0 | 0 | 0 | 110 | 2000 |
| 17 | Toxicology and Industrial Health | 15004 | 1429 | 49 | 10,5 | 1990 | 6 | 1,710 | 1,591 | 3 | 0 | 0 | 115 | 1985 |
| 18 | Journal of Environmental Science and Health Part B-Pesticides Food Contaminants and Agricultural Wastes | 13210 | 1901 | 34 | 6,95 | 1990 | 25 | 1,234 | 1,129 | 0 | 0 | 0 | 122 | 1976 |
| 19 | Environmental Geochemistry and Health | 12095 | 1093 | 42 | 11,07 | 1990 | 12 | 2,573 | 2,534 | 0 | 0 | 0 | 128 | 1979 |
| 20 | Industrial Health | 10652 | 1444 | 35 | 7,38 | 1990 | 28 | 1,045 | 1,132 | 0 | 0 | 0 | 140 | 1963 |
| 21 | Journal of Toxicology and Environmental Health-Part B-Critical Reviews | 9483 | 285 | 53 | 33,27 | 1998 | 1 | 5,146 | 5,422 | 0 | 0 | 0 | 148 | 1998 |
| 22 | Journal of Occupational Health | 8701 | 1128 | 36 | 7,71 | 1997 | 39 | 1,096 | 1,792 | 0 | 0 | 0 | 157 | 1996 |
| 23 | Journal of Occupational Health Psychology | 7922 | 371 | 46 | 21,35 | 2004 | 9 | 2,178 | 3,017 | 0 | 0 | 0 | 161 | 1996 |
| 24 | Annals of Agricultural and Environmental Medicine | 7614 | 1065 | 32 | 7,15 | 2000 | 7 | 3,06 | - | 0 | 0 | 0 | 164 | 1994 |
| 25 | Journal of Exposure Science and Environmental Epidemiology | 7105 | 641 | 37 | 11,08 | 2006 | 16 | 3,050 | 2,917 | 0 | 0 | 0 | 171 | 1991 |
| 26 | Biomedical and Environmental Sciences | 7084 | 1223 | 30 | 5,79 | 1998 | 11 | 1,257 | 1,46 | 0 | 0 | 0 | 172 | 1988 |
| 27 | Environmental Health | 6798 | 718 | 36 | 9,47 | 2007 | 6 | 2,713 | 3,259 | 0 | 0 | 0 | 175 | 2002 |
| 28 | Journal of Occupational and Environmental Hygiene | 6704 | 1090 | 33 | 6,15 | 2004 | 1 | 1,207 | 1,376 | 0 | 0 | 0 | 176 | 2004 |
| 29 | International Journal of Environmental Health Research | 5140 | 638 | 28 | 8,06 | 1992 | 2 | 1,513 | 1,634 | 0 | 0 | 0 | 190 | 1991 |
| 30 | Indoor and Built Environment | 5093 | 1011 | 24 | 5,04 | 1996 | 5 | 1,716 | 1,494 | 0 | 0 | 0 | 191 | 1992 |
| 31 | International Journal of Occupational and Environmental Health | 4585 | 679 | 26 | 6,75 | 2002 | 8 | 1,099 | 1,077 | 0 | 0 | 0 | 199 | 1995 |
| 32 | Wilderness & Environmental Medicine | 4442 | 1093 | 23 | 4,06 | 1995 | 6 | 0,790 | 0,925 | 0 | 0 | 0 | 200 | 1990 |
| 33 | Journal of Environmental Health | 3074 | 1268 | 19 | 2,42 | 1990 | 53 | 0,893 | 1,139 | 0 | 0 | 0 | 221 | 1938 |
| 34 | International Journal of Circumpolar Health | 2110 | 591 | 19 | 3,57 | 2006 | 65 | 1,299 | 1,550 | 0 | 0 | 0 | 235 | 1972 |
| 35 | Archives of Environmental & Occupational Health | 1429 | 306 | 17 | 4,67 | 2005 | 60 | 0,617 | 0,966 | 0 | 0 | 0 | 255 | 1960 |
| 36 | International Journal of Occupational Medicine and Environmental Health | 1321 | 426 | 16 | 3,10 | 2008 | 21 | 1,094 | 1,400 | 0 | 0 | 0 | 258 | 1988 |
| 37 | Noise & Health | 1255 | 306 | 16 | 4,1 | 2008 | 10 | 1,430 | 1,641 | 0 | 0 | 0 | 261 | 1999 |
| 38 | Arhiv Za Higijenu Rada I Toksikologiju-Archives of Industrial Hygiene and Toxicology | 1095 | 362 | 15 | 3,02 | 2008 | 59 | 0,727 | 0,980 | 0 | 0 | 0 | 270 | 1919 |
| 39 | International Journal of Occupational Safety and Ergonomics | 839 | 413 | 12 | 2,03 | 2006 | 12 | 0,253 | 0,595 | 0 | 0 | 0 | 284 | 1995 |
| 40 | Medicina del Lavoro | 631 | 429 | 9 | 1,47 | 2007 | 98 | 0,482 | 0,381 | 0 | 0 | 0 | 295 | 1901 |
| 41 | Journal of Agromedicine | 617 | 246 | 10 | 2,51 | 2009 | 14 | 0,924 | N/A | 0 | 0 | 0 | 296 | 1994 |
| 42 | Herd-Health Environments Research & Design Journal | 517 | 250 | 9 | 2,07 | 2007 | 1 | 0,214 | 0,446 | 0 | 0 | 0 | 307 | 2007 |
| 43 | Journal of Occupational Medicine and Toxicology | 501 | 165 | 10 | 3,04 | 2010 | 5 | 1,226 | N/A | 0 | 0 | 0 | 308 | 2006 |
| 44 | Disaster Prevention and Management | 324 | 235 | 7 | 1,38 | 2009 | 18 | 0,380 | N/A | 0 | 0 | 0 | 326 | 1992 |
| 45 | Radioprotection | 240 | 267 | 6 | 0,9 | 1990 | 25 | 0,596 | 0,854 | 0 | 0 | 0 | 335 | 1966 |
| 46 | Archives Des Maladies Professionnelles Et de L'Environnement | 206 | 555 | 4 | 0,37 | 2007 | 68 | 0,090 | 0,098 | 0 | 0 | 0 | 339 | 1940 |
| 47 | Environmental Health and Preventive Medicine | 100 | 117 | 5 | 0,85 | 2013 | 18 | N/A | N/A | 0 | 0 | 0 | 355 | 1996 |

Table S3. Health Management and Economics

| R | Journal Name | TC | TP | H | TC/TP | Year | Volume | IF | IF5 | T50 | T200 | T200* | GR | First Year |
| --- | --- | --- | --- | --- | --- | --- | --- | --- | --- | --- | --- | --- | --- | --- |
| 1 | Medical Care | 162015 | 3890 | 145 | 41,65 | 1990 | 28 | 2,941 | 3,714 | 31 | 16 | 10 | 9 | 1963 |
| 2 | Risk Analysis | 50164 | 2716 | 85 | 18,47 | 1990 | 10 | 1,974 | 2,546 | 5 | 2 | 1 | 38 | 1981 |
| 3 | Journal of Health Economics | 39818 | 1400 | 85 | 28,44 | 1990 | 9 | 2,254 | 3,159 | 5 | 3 | 1 | 49 | 1982 |
| 4 | Pharmacoeconomics | 37078 | 2163 | 69 | 17,04 | 1993 | 4 | 3,338 | 3,509 | 2 | 0 | 0 | 57 | 1992 |
| 5 | Medical Decision Making | 34695 | 1511 | 80 | 22,96 | 1990 | 10 | 2,698 | 3,083 | 5 | 1 | 1 | 59 | 1981 |
| 6 | Health Economics | 32797 | 1643 | 72 | 19,96 | 1994 | 3 | 2,137 | 2,570 | 2 | 0 | 0 | 65 | 1992 |
| 7 | BMC Health Services Research | 25343 | 3474 | 49 | 7,3 | 2001 | 1 | 1,659 | 2,188 | 0 | 0 | 0 | 72 | 2001 |
| 8 | American Journal of Managed Care | 24099 | 2483 | 57 | 9,71 | 1997 | 3 | 2,166 | 2,688 | 0 | 0 | 0 | 76 | 1995 |
| 9 | Inquiry-The Journal of Health Care Organization Provision and Financing | 9704 | 711 | 43 | 13,65 | 1990 | 97 | 0,564 | 0,793 | 0 | 0 | 0 | 146 | 1964 |
| 10 | Health Care Management Review | 6392 | 702 | 32 | 9,11 | 1994 | 19 | 1,642 | 2,071 | 0 | 0 | 0 | 179 | 1976 |
| 11 | American Journal of Medical Quality | 5264 | 698 | 30 | 7,5 | 1999 | 14 | 1,776 | 1,821 | 0 | 0 | 0 | 189 | 1986 |
| 12 | Journal of Managed Care Pharmacy | 4630 | 616 | 31 | 7,52 | 2006 | 12 | 2,682 | 2,858 | 0 | 0 | 0 | 198 | 1995 |
| 13 | Journal of Public Health Management and Practice | 3661 | 865 | 21 | 4,23 | 2005 | 11 | 0,84 | 1,198 | 0 | 0 | 0 | 211 | 1995 |
| 14 | International Journal of Health Planning and Management | 3470 | 512 | 26 | 6,78 | 1994 | 9 | 0,971 | 1,029 | 0 | 0 | 0 | 213 | 1985 |
| 15 | Economics & Human Biology | 3326 | 348 | 29 | 9,56 | 2006 | 4 | 2,461 | 2,878 | 0 | 0 | 0 | 217 | 2003 |
| 16 | Journal of Healthcare Management | 2919 | 514 | 24 | 5,68 | 1998 | 43 | 0,96 | 0,977 | 0 | 0 | 0 | 224 | 2007 |
| 17 | European Journal of Health Economics | 2403 | 495 | 19 | 4,85 | 2007 | 8 | 1,913 | 1,865 | 0 | 0 | 0 | 230 | 2000 |
| 18 | Australian Health Review | 2098 | 669 | 17 | 3,14 | 2007 | 31 | 1,000 | 1,127 | 0 | 0 | 0 | 236 | 1978 |
| 19 | Human Resources for Health | 2048 | 371 | 20 | 5,52 | 2008 | 6 | 1,922 | 2,392 | 0 | 0 | 0 | 237 | 2003 |
| 20 | International Journal for Equity in Health | 1761 | 458 | 16 | 3,84 | 2008 | 7 | 1,589 | 1,925 | 0 | 0 | 0 | 247 | 2002 |
| 21 | Expert Review of Pharmacoeconomics & Outcomes Research | 1364 | 352 | 17 | 3,88 | 2010 | 10 | 1,870 | N/A | 0 | 0 | 0 | 256 | 2001 |
| 22 | Research in Social & Administrative Pharmacy | 1178 | 355 | 13 | 3,32 | 2008 | 4 | 1,202 | 1,396 | 0 | 0 | 0 | 267 | 2005 |
| 23 | Population Health Management | 1026 | 317 | 13 | 3,24 | 2008 | 11 | 1,347 | 1,369 | 0 | 0 | 0 | 272 | 1998 |
| 24 | Therapeutics and Clinical Risk Management | 965 | 338 | 13 | 2,86 | 2010 | 6 | 1,343 | N/A | 0 | 0 | 0 | 277 | 2005 |
| 25 | Health Care Management Science | 951 | 220 | 14 | 4,32 | 2008 | 11 | 0,871 | 1,025 | 0 | 0 | 0 | 279 | 1998 |
| 26 | Sciences Sociales Et Sante | 635 | 314 | 10 | 2,02 | 1994 | 12 | 0,194 | 0,219 | 0 | 0 | 0 | 294 | 1983 |
| 27 | International Journal of Health Care Finance & Economics | 362 | 127 | 8 | 2,85 | 2008 | 8 | 0,71 | 0,612 | 0 | 0 | 0 | 320 | 2001 |
| 28 | Health Information Management Journal | 337 | 111 | 8 | 3,04 | 2007 | 36 | 0,704 | 0,824 | 0 | 0 | 0 | 323 | 1930 |
| 29 | Journal of Comparative Effectiveness Research | 163 | 149 | 4 | 1,09 | 2012 | 1 | N/A | N/A | 0 | 0 | 0 | 344 | 2012 |

Table S4. Health Promotion and Health Behavior

| R | Journal Name | TC | TP | H | TC/TP | Year | Volume | IF | IF5 | T50 | T200 | T200* | GR | First Year |
| --- | --- | --- | --- | --- | --- | --- | --- | --- | --- | --- | --- | --- | --- | --- |
| 1 | American Journal of Preventive Medicine | 115942 | 3947 | 133 | 29,37 | 1990 | 6 | 4,281 | 5,092 | 13 | 5 | 5 | 12 | 1985 |
| 2 | Preventive Medicine | 111066 | 4186 | 122 | 26,53 | 1990 | 19 | 2,932 | 3,917 | 4 | 3 | 3 | 14 | 1972 |
| 3 | Journal of Adolescent Health | 78449 | 3676 | 96 | 21,34 | 1990 | 11 | 2,748 | 3,753 | 2 | 0 | 0 | 23 | 1980 |
| 4 | Patient Education and Counseling | 59813 | 3611 | 83 | 16,56 | 1990 | 15 | 2,598 | 3,158 | 2 | 0 | 0 | 33 | 1978 |
| 5 | Medical Education | 50964 | 3253 | 79 | 15,67 | 1990 | 24 | 3,617 | 3,963 | 0 | 0 | 0 | 37 | 1966 |
| 6 | Journal of Health and Social Behavior | 45820 | 728 | 107 | 62,94 | 1990 | 31 | 2,951 | 4,457 | 8 | 4 | 4 | 44 | 1960 |
| 7 | Supportive Care in Cancer | 39781 | 3384 | 62 | 11,76 | 1993 | 1 | 2,495 | 2,845 | 0 | 0 | 0 | 50 | 1993 |
| 8 | AIDS Care-Psychological and Socio-Medical Aspects of AIDS/HIV | 39017 | 2795 | 66 | 13,96 | 1992 | 4 | 2,194 | 2,454 | 1 | 1 | 1 | 53 | 1989 |
| 9 | American Journal of Community Psychology | 33534 | 1259 | 84 | 26,64 | 1990 | 18 | 1,968 | 2,888 | 1 | 1 | 0 | 63 | 1973 |
| 10 | Tobacco Control | 32078 | 1641 | 74 | 19,55 | 1998 | 7 | 5,150 | 4,532 | 1 | 0 | 0 | 66 | 1992 |
| 11 | Health Education Research | 31136 | 1594 | 73 | 19,53 | 1991 | 6 | 1,944 | 2,508 | 0 | 0 | 0 | 67 | 1986 |
| 12 | Qualitative Health Research | 28115 | 1864 | 58 | 15,08 | 1995 | 5 | 1,441 | - | 1 | 1 | 0 | 70 | 1991 |
| 13 | Psychology & Health | 24761 | 1407 | 57 | 17,6 | 1992 | 7 | 2,255 | 2,107 | 5 | 1 | 1 | 74 | 1987 |
| 14 | Medical Teacher | 24402 | 3370 | 52 | 7,24 | 1990 | 12 | 2,045 | 2,170 | 1 | 1 | 1 | 75 | 1979 |
| 15 | Sociology of Health & Illness | 23121 | 1134 | 65 | 20,39 | 1990 | 12 | 2,014 | 2,62 | 1 | 0 | 0 | 77 | 1979 |
| 16 | AIDS and Behavior | 22636 | 1863 | 55 | 12,15 | 2003 | 7 | 3,312 | 3,977 | 0 | 0 | 0 | 81 | 1997 |
| 17 | American Journal of Health Promotion | 21372 | 1072 | 63 | 19,94 | 1995 | 9 | 1,762 | 2,389 | 4 | 2 | 2 | 83 | 1986 |
| 18 | AIDS Education and Prevention | 20901 | 1123 | 57 | 18,61 | 1992 | 4 | 1,505 | 2,298 | 0 | 0 | 0 | 86 | 1989 |
| 19 | Journal of School Health | 20436 | 1887 | 56 | 10,83 | 1990 | 60 | 1,659 | 2,132 | 0 | 0 | 0 | 90 | 1930 |
| 20 | AIDS Patient Care and STDs | 20210 | 1622 | 49 | 12,46 | 1996 | 10 | 3,576 | 3,255 | 0 | 0 | 0 | 93 | 1987 |
| 21 | Health Education & Behavior | 20163 | 962 | 57 | 20,96 | 1997 | 24 | 1,825 | 2,507 | 3 | 1 | 1 | 94 | 1957 |
| 22 | Journal of Community Psychology | 16914 | 1197 | 58 | 14,13 | 1990 | 18 | 0,832 | 1,488 | 0 | 0 | 0 | 105 | 1973 |
| 23 | Community Mental Health Journal | 15044 | 1391 | 49 | 10,82 | 1990 | 26 | 1,146 | 1,422 | 0 | 0 | 0 | 113 | 1965 |
| 24 | Studies in Family Planning | 13429 | 723 | 52 | 18,57 | 1990 | 21 | 1,638 | 2,529 | 0 | 0 | 0 | 117 | 1963 |
| 25 | Women & Health | 13258 | 1065 | 44 | 12,45 | 1990 | 16 | 1,194 | 1,305 | 0 | 0 | 0 | 121 | 1975 |
| 26 | Health Promotion International | 12412 | 914 | 49 | 13,58 | 1994 | 9 | 1,736 | 2,332 | 1 | 0 | 0 | 125 | 1986 |
| 27 | Injury Prevention | 12131 | 1044 | 41 | 11,62 | 2002 | 8 | 1,941 | 2,036 | 0 | 0 | 0 | 126 | 1995 |
| 28 | Diabetes Educator | 12128 | 1098 | 44 | 11,05 | 1993 | 19 | 1,919 | 2,414 | 0 | 0 | 0 | 127 | 1975 |
| 29 | American Journal of Health Behavior | 12027 | 1198 | 43 | 10,04 | 1996 | 20 | 1,137 | 1,69 | 0 | 0 | 0 | 129 | 1996 |
| 30 | Future of Children | 11604 | 401 | 52 | 28,94 | 1995 | 5 | 2,273 | 4,059 | 1 | 0 | 0 | 134 | 1991 |
| 31 | Health & Social Care in The Community | 10049 | 1132 | 36 | 8,88 | 1994 | 2 | 1,151 | 1,54 | 0 | 0 | 0 | 142 | 1993 |
| 32 | Health Communication | 9163 | 883 | 39 | 10,38 | 1994 | 6 | 1,276 | 1,563 | 0 | 0 | 0 | 151 | 1989 |
| 33 | Womens Health Issues | 8314 | 952 | 34 | 8,73 | 1995 | 5 | 1,735 | 1,870 | 0 | 0 | 0 | 158 | 1990 |
| 34 | Evaluation & The Health Professions | 7853 | 671 | 39 | 11,7 | 1990 | 13 | 1,672 | 1,710 | 0 | 0 | 0 | 162 | 1978 |
| 35 | Prevention Science | 7400 | 458 | 39 | 16,16 | 2004 | 5 | 2,827 | 3,413 | 0 | 0 | 0 | 167 | 2000 |
| 36 | Teaching and Learning in Medicine | 6520 | 927 | 30 | 7,03 | 1996 | 8 | 1,118 | 1,173 | 0 | 0 | 0 | 178 | 1989 |
| 37 | Advances in Health Sciences Education | 6313 | 594 | 32 | 10,63 | 1996 | 1 | 2,705 | 2,976 | 0 | 0 | 0 | 180 | 1996 |
| 38 | Journal of Behavioral Health Services & Research | 6212 | 627 | 32 | 9,91 | 1998 | 25 | 1,026 | 1,241 | 0 | 0 | 0 | 181 | 1972 |
| 39 | Reproductive Health Matters | 5992 | 605 | 34 | 9,9 | 2000 | 8 | 1,740 | 1,976 | 0 | 0 | 0 | 184 | 1993 |
| 40 | Journal of Cancer Education | 4841 | 1045 | 24 | 4,63 | 2000 | 15 | 1,054 | 0,978 | 0 | 0 | 0 | 195 | 1986 |
| 41 | Family & Community Health | 4686 | 650 | 27 | 7,21 | 1994 | 17 | 0,855 | 1,245 | 0 | 0 | 0 | 197 | 1978 |
| 42 | European Journal of Contraception and Reproductive Health Care | 4188 | 693 | 24 | 6,04 | 1999 | 4 | 1,835 | 1,667 | 0 | 0 | 0 | 204 | 1996 |
| 43 | Health Risk & Society | 4149 | 429 | 27 | 9,67 | 2000 | 2 | 1,653 | 1,900 | 0 | 0 | 0 | 206 | 1999 |
| 44 | Journal of Continuing Education in the Health Professions | 3899 | 393 | 26 | 9,92 | 2005 | 25 | 1,190 | 1,729 | 1 | 1 | 1 | 209 | 1981 |
| 45 | Sexual Health | 3134 | 586 | 24 | 5,35 | 2007 | 4 | 1,576 | 1,796 | 0 | 0 | 0 | 219 | 2004 |
| 46 | Journal of Religion & Health | 2942 | 1044 | 20 | 2,82 | 1990 | 29 | 0,945 | 1,155 | 0 | 0 | 0 | 223 | 1961 |
| 47 | Traffic Injury Prevention | 2865 | 668 | 19 | 4,29 | 2008 | 9 | 1,286 | 1,556 | 0 | 0 | 0 | 225 | 1999 |
| 48 | Preventing Chronic Disease | 2834 | 949 | 20 | 2,99 | 2009 | 6 | 1,956 | N/A | 0 | 0 | 0 | 227 | 2004 |
| 49 | Work-A Journal of Prevention Assessment & Rehabilitation | 2705 | 2091 | 15 | 1,29 | 2008 | 30 | 0,169 | 0,357 | 0 | 0 | 0 | 228 | 1990 |
| 50 | Journal of Interprofessional Care | 2215 | 565 | 21 | 3,92 | 2008 | 22 | 1,362 | 1,466 | 0 | 0 | 0 | 232 | 1986 |
| 51 | Australian Journal of Rural Health | 2206 | 420 | 19 | 5,25 | 2007 | 15 | 1,343 | 1,486 | 0 | 0 | 0 | 233 | 1992 |
| 52 | Health Care for Women International | 1294 | 460 | 14 | 2,81 | 2008 | 29 | 0,696 | 1,010 | 0 | 0 | 0 | 259 | 1978 |
| 53 | American Journal of Mens Health | 1227 | 348 | 14 | 3,53 | 2007 | 1 | 1,269 | 1,243 | 0 | 0 | 0 | 263 | 2007 |
| 54 | Journal of Primary Prevention | 1061 | 203 | 16 | 5,23 | 2008 | 29 | 1,250 | 1,701 | 0 | 0 | 0 | 271 | 1980 |
| 55 | Disability and Health Journal | 994 | 269 | 13 | 3,7 | 2008 | 1 | 1,500 | 1,519 | 0 | 0 | 0 | 275 | 2008 |
| 56 | BMC Womens Health | 881 | 338 | 12 | 2,61 | 2010 | 10 | 1,657 | N/A | 0 | 0 | 0 | 282 | 2001 |
| 57 | Health Promotion Journal of Australia | 841 | 289 | 12 | 2,91 | 2008 | 19 | 1,089 | 1,294 | 0 | 0 | 0 | 283 | 1990 |
| 58 | AJAR-African Journal of AIDS Research | 774 | 308 | 11 | 2,51 | 2007 | 6 | 0,608 | 0,671 | 0 | 0 | 0 | 286 | 2002 |
| 59 | Health Sociology Review | 774 | 251 | 11 | 3,08 | 2007 | 16 | 0,456 | 0,848 | 0 | 0 | 0 | 287 | 1991 |
| 60 | Patient-Patient Centered Outcomes Research | 760 | 189 | 10 | 4,02 | 2008 | 1 | 1,957 | 1,603 | 0 | 0 | 0 | 288 | 2008 |
| 61 | Families Systems & Health | 749 | 173 | 15 | 4,33 | 2009 | 27 | 1,039 | N/A | 0 | 0 | 0 | 290 | 1983 |
| 62 | International Journal of Injury Control and Safety Promotion | 666 | 282 | 10 | 2,36 | 2008 | 15 | 0,544 | 0,821 | 0 | 0 | 0 | 292 | 1994 |
| 63 | International Perspectives on Sexual and Reproductive Health | 611 | 138 | 13 | 4,43 | 2009 | 35 | 1,938 | 1,687 | 0 | 0 | 0 | 297 | 1975 |
| 64 | Health Education Journal | 560 | 375 | 9 | 1,49 | 2007 | 66 | 0,694 | 1,209 | 0 | 0 | 0 | 301 | 1943 |
| 65 | Palliative & Supportive Care | 543 | 224 | 10 | 2,42 | 2010 | 8 | 1,211 | N/A | 0 | 0 | 0 | 304 | 2003 |
| 66 | Current Opinion in Supportive and Palliative Care | 366 | 185 | 9 | 1,98 | 2012 | 6 | N/A | N/A | 0 | 0 | 0 | 319 | 2007 |
| 67 | Sexual & Reproductive Healthcare | 345 | 140 | 9 | 2,46 | 2010 | 1 | 1,250 | 1,159 | 0 | 0 | 0 | 321 | 2010 |
| 68 | International Journal of Sexual Health | 339 | 161 | 8 | 2,11 | 2008 | 20 | 0,688 | 0,735 | 0 | 0 | 0 | 322 | 1988 |
| 69 | Global Health Promotion | 301 | 186 | 7 | 1,62 | 2010 | 17 | 0,522 | N/A | 0 | 0 | 0 | 330 | 1994 |
| 70 | Progress in Community Health Partnerships-Research Education and Action | 206 | 187 | 6 | 1,1 | 2011 | 5 | 0,793 | N/A | 0 | 0 | 0 | 340 | 2007 |
| 71 | Sex Education-Sexuality Society and Learning | 111 | 151 | 4 | 0,74 | 2012 | 12 | N/A | N/A | 0 | 0 | 0 | 354 | 2001 |
| 72 | Journal of Correctional Health Care | 59 | 80 | 4 | 0,74 | 2012 | 18 | N/A | N/A | 0 | 0 | 0 | 357 | 1994 |
| 73 | Health and Human Rights | 31 | 84 | 3 | 0,37 | 2011 | 13 | 0,655 | N/A | 0 | 0 | 0 | 359 | 1994 |

Table S5. Epidemiology

| R | Journal Name | TC | TP | H | TC/TP | Year | Volume | IF | IF5 | T50 | T200 | T200* | GR | First Year |
| --- | --- | --- | --- | --- | --- | --- | --- | --- | --- | --- | --- | --- | --- | --- |
| 1 | American Journal of Epidemiology | 357243 | 8162 | 211 | 43,77 | 1990 | 131 | 4,975 | 6,067 | 11 | 8 | 49 | 1 | 1921 |
| 2 | Cancer Epidemiology Biomarkers & Prevention | 207614 | 6073 | 147 | 34,19 | 1991 | 1 | 4,324 | 4,647 | 2 | 1 | 0 | 5 | 1991 |
| 3 | Journal of Clinical Epidemiology | 168450 | 4502 | 167 | 37,42 | 1990 | 43 | 5,478 | 5,898 | 17 | 16 | 2 | 7 | 1955 |
| 4 | Statistics in Medicine | 148692 | 6505 | 140 | 22,86 | 1990 | 9 | 2,037 | 2,828 | 0 | 0 | 6 | 10 | 1982 |
| 5 | International Journal of Epidemiology | 140691 | 4335 | 134 | 32,45 | 1990 | 19 | 9,197 | 8,000 | 3 | 0 | 3 | 11 | 1972 |
| 6 | Journal of Epidemiology and Community Health | 99343 | 3939 | 117 | 25,22 | 1990 | 44 | 3,294 | 3,667 | 2 | 2 | 3 | 15 | 1947 |
| 7 | Epidemiology | 93837 | 3124 | 126 | 30,04 | 1991 | 2 | 6,178 | 6,894 | 1 | 1 | 0 | 16 | 1990 |
| 8 | Infection Control and Hospital Epidemiology | 86969 | 4588 | 100 | 18,96 | 1990 | 11 | 3,938 | 4,423 | 5 | 3 | 0 | 18 | 1980 |
| 9 | Cancer Causes & Control | 83834 | 3003 | 112 | 27,92 | 1990 | 1 | 2,961 | 3,434 | 1 | 1 | 0 | 21 | 1990 |
| 10 | Epidemiology and Infection | 73121 | 4117 | 85 | 17,76 | 1990 | 104 | 2,491 | 2,671 | 0 | 0 | 0 | 25 | 1901 |
| 11 | European Journal of Epidemiology | 46372 | 2896 | 71 | 16,01 | 1990 | 6 | 5,147 | 4,245 | 1 | 1 | 0 | 43 | 1985 |
| 12 | Annals of Epidemiology | 43089 | 2002 | 83 | 21,52 | 1996 | 6 | 2,145 | 2,895 | 0 | 0 | 0 | 48 | 1990 |
| 13 | Genetic Epidemiology | 38300 | 2040 | 77 | 18,77 | 1990 | 7 | 2,951 | 3,489 | 1 | 1 | 1 | 54 | 1984 |
| 14 | Epidemiologic Reviews | 34630 | 456 | 93 | 75,94 | 1990 | 12 | 7,333 | 12,344 | 4 | 3 | 5 | 60 | 1979 |
| 15 | Community Dentistry and Oral Epidemiology | 33089 | 1726 | 66 | 19,17 | 1990 | 18 | 1,944 | 2,491 | 0 | 0 | 0 | 64 | 1973 |
| 16 | Neuroepidemiology | 24773 | 1314 | 62 | 18,85 | 1990 | 9 | 2,476 | 2,863 | 0 | 0 | 0 | 73 | 1982 |
| 17 | Paediatric and Perinatal Epidemiology | 21249 | 1228 | 59 | 17,3 | 1994 | 8 | 2,811 | 2,796 | 0 | 0 | 0 | 84 | 1987 |
| 18 | Journal of Urban Health-Bulletin of The New York Academy of Medicine | 18231 | 1274 | 54 | 14,31 | 1998 | 75 | 1,943 | 2,517 | 0 | 0 | 0 | 99 | 1925 |
| 19 | Statistical Methods in Medical Research | 11801 | 490 | 44 | 24,08 | 1999 | 8 | 2,957 | 3,155 | 2 | 2 | 0 | 131 | 1992 |
| 20 | BMC Medical Research Methodology | 8936 | 979 | 39 | 9,13 | 2007 | 7 | 2,168 | 3,024 | 0 | 0 | 0 | 153 | 2001 |
| 21 | Revue D Epidemiologie Et de Sante Publique | 7563 | 1391 | 28 | 5,44 | 1990 | 38 | 0,656 | 1,049 | 0 | 0 | 0 | 165 | 1953 |
| 22 | Journal of Epidemiology | 6929 | 679 | 34 | 10,2 | 2003 | 13 | 2,862 | 2,498 | 0 | 0 | 0 | 173 | 1991 |
| 23 | Cancer Epidemiology | 4162 | 739 | 23 | 5,63 | 2009 | 33 | 2,558 | 2,468 | 0 | 0 | 0 | 205 | 1981 |
| 24 | Epidemiologia & Prevenzione | 594 | 434 | 8 | 1,37 | 2007 | 31 | 1,456 | 0,948 | 0 | 0 | 0 | 299 | 1979 |
| 25 | Chronic Diseases and Injuries in Canada | 217 | 101 | 7 | 2,15 | 2011 | 31 | 1,222 | 1,222 | 0 | 0 | 0 | 338 | 1980 |

Table S6. Health Policy and Services

| R | Journal Name | TC | TP | H | TC/TP | Year | Volume | IF | IF5 | T50 | T200 | T200* | GR | Frst Year | |
| --- | --- | --- | --- | --- | --- | --- | --- | --- | --- | --- | --- | --- | --- | --- | --- |
| 1 | Health Affairs | 85858 | 5495 | 106 | 15,62 | 1990 | 9 | 4,321 | 4,402 | 11 | 1 | 1 | 19 | | 1981 |
| 2 | Quality of Life Research | 65856 | 2697 | 102 | 24,42 | 1993 | 2 | 2,864 | 3,270 | 15 | 3 | 3 | 28 | | 1992 |
| 3 | Health Services Research | 44904 | 1997 | 82 | 22,49 | 1990 | 24 | 2,491 | 2,772 | 4 | 0 | 0 | 46 | | 1967 |
| 4 | Health Policy | 29530 | 1646 | 53 | 11,16 | 1990 | 14 | 1,725 | 1,923 | 1 | 1 | 1 | 68 | | 1980 |
| 5 | Milbank Quarterly | 22965 | 605 | 69 | 37,96 | 1990 | 68 | 5,391 | 6,513 | 8 | 3 | 3 | 78 | | 1923 |
| 6 | Health Policy and Planning | 20646 | 1306 | 56 | 15,81 | 1991 | 6 | 3,442 | 3,703 | 1 | 0 | 0 | 89 | | 1986 |
| 7 | International Journal for Quality in Health Care | 18340 | 1299 | 54 | 14,12 | 1995 | 7 | 1,584 | 2,296 | 1 | 0 | 0 | 98 | | 1989 |
| 8 | Value in Health | 18129 | 1403 | 53 | 12,92 | 2002 | 5 | 2,891 | 3,174 | 3 | 0 | 0 | 100 | | 1998 |
| 9 | Community Mental Health Journal | 15045 | 1391 | 49 | 10,82 | 1990 | 26 | 1,146 | 1,422 | 0 | 0 | 0 | 113 | | 1965 |
| 10 | Medical Care Research and Review | 13328 | 636 | 55 | 20,96 | 1995 | 52 | 2,600 | 3,638 | 2 | 0 | 0 | 119 | | 1944 |
| 11 | Hastings Center Report | 11744 | 2179 | 47 | 5,39 | 1990 | 20 | 1,080 | 1,131 | 0 | 0 | 0 | 132 | | 1971 |
| 12 | International Journal of Health Services | 11664 | 1102 | 46 | 10,58 | 1990 | 20 | 0,988 | 1,236 | 2 | 0 | 0 | 133 | | 1971 |
| 13 | Health and Quality of Life Outcomes | 11595 | 1199 | 44 | 9,67 | 2006 | 4 | 2,099 | 3,152 | 0 | 0 | 0 | 135 | | 2003 |
| 14 | Journal of Aging and Health | 11313 | 857 | 45 | 13,2 | 1995 | 7 | 1,832 | 2,057 | 0 | 0 | 0 | 136 | | 1989 |
| 15 | Journal of Health Politics Policy and Law | 10243 | 1023 | 41 | 10,01 | 1990 | 15 | 0,962 | 1,169 | 0 | 0 | 0 | 141 | | 1976 |
| 16 | Journal of Health Care for The Poor and Underserved | 9865 | 1466 | 35 | 6,73 | 1995 | 6 | 0,902 | 1,394 | 0 | 0 | 0 | 144 | | 1990 |
| 17 | Journal of Community Health | 9787 | 1247 | 39 | 7,85 | 1994 | 19 | 1,573 | 1,765 | 0 | 0 | 0 | 145 | | 1975 |
| 18 | Psychology Public Policy and Law | 9354 | 508 | 45 | 18,41 | 1995 | 1 | 1,723 | 2,697 | 2 | 0 | 0 | 149 | | 1995 |
| 19 | Journal of Rural Health | 8759 | 943 | 37 | 9,29 | 1996 | 12 | 1,771 | 1,844 | 0 | 0 | 0 | 156 | | 1985 |
| 20 | Gesundheitswesen | 5518 | 1610 | 23 | 3,43 | 2000 | 62 | 0,624 | 0,621 | 0 | 0 | 0 | 187 | | 1946 |
| 21 | Health Expectations | 4969 | 434 | 33 | 11,45 | 2004 | 7 | 2,852 | 3,016 | 0 | 0 | 0 | 193 | | 1998 |
| 22 | Journal of Public Health Policy | 4217 | 531 | 30 | 7,94 | 1994 | 15 | 1,750 | 2,189 | 0 | 0 | 0 | 203 | | 1980 |
| 23 | Administration and Policy in Mental Health and Mental Health Services Research | 4053 | 429 | 27 | 9,45 | 1993 | 21 | 3,442 | 3,254 | 0 | 0 | 0 | 208 | | 1973 |
| 24 | Cambridge Quarterly of Healthcare Ethics | 3601 | 865 | 21 | 4,16 | 1994 | 3 | 0,584 | 0,589 | 0 | 0 | 0 | 212 | | 1992 |
| 25 | BMJ Quality & Safety | 3237 | 554 | 22 | 5,84 | 2011 | 20 | 3,281 | 3,308 | 0 | 0 | 0 | 218 | | 1992 |
| 26 | Health Care Analysis | 2850 | 575 | 23 | 4,96 | 1994 | 2 | 1,078 | 1,289 | 0 | 0 | 0 | 226 | | 1993 |
| 27 | Journal of Health Services Research & Policy | 1761 | 310 | 19 | 5,68 | 2008 | 13 | 2,087 | 2,055 | 0 | 0 | 0 | 248 | | 1996 |
| 28 | Journal of Mental Health Policy and Economics | 1233 | 158 | 14 | 7,8 | 2005 | 8 | 1,406 | 1,325 | 0 | 0 | 0 | 262 | | 1998 |
| 29 | Health Economics Policy and Law | 1191 | 211 | 16 | 5,64 | 2006 | 1 | 1,593 | 1,760 | 0 | 0 | 0 | 266 | | 2006 |
| 30 | Disability and Health Journal | 994 | 269 | 13 | 3,7 | 2008 | 1 | 1,500 | 1,519 | 0 | 0 | 0 | 275 | | 2008 |
| 31 | Health Research Policy and Systems | 921 | 243 | 12 | 3,79 | 2010 | 8 | 1,860 | N/A | 0 | 0 | 0 | 280 | | 2003 |
| 32 | Journal of Policy and Practice in Intellectual Disabilities | 751 | 235 | 12 | 3,2 | 2008 | 5 | 0,629 | 1,041 | 0 | 0 | 0 | 289 | | 2004 |
| 33 | Sahara J-Journal of Social Aspects of HIV-AIDS | 524 | 177 | 11 | 2,96 | 2008 | 5 | 0,393 | 1,080 | 0 | 0 | 0 | 306 | | 2004 |
| 34 | BMC Palliative Care | 327 | 148 | 8 | 2,21 | 2011 | 10 | 1,787 | N/A | 0 | 0 | 0 | 324 | | 2002 |
| 35 | Social Work in Public Health | 314 | 267 | 6 | 1,18 | 2009 | 24 | 0,333 | N/A | 0 | 0 | 0 | 328 | | 1989 |
| 36 | Journal of Patient Safety | 292 | 135 | 8 | 2,16 | 2011 | 7 | 0,877 | N/A | 0 | 0 | 0 | 331 | | 2005 |
| 37 | Journal for Healthcare Quality | 136 | 109 | 6 | 1,25 | 2012 | 34 | N/A | N/A | 0 | 0 | 0 | 348 | | 1980 |
| 38 | Asian Journal of WTO & International Health Law and Policy | 131 | 142 | 5 | 0,92 | 2007 | 2 | 0,194 | 0,226 | 0 | 0 | 0 | 349 | | 2006 |
| 39 | Israel Journal of Health Policy Research | 129 | 72 | 4 | 1,79 | 2012 | 1 | 1,250 | 1,250 | 0 | 0 | 0 | 350 | | 2012 |
| 40 | International Journal of Integrated Care | 16 | 193 | 2 | 0,08 | 2010 | 10 | 1,261 | N/A | 0 | 0 | 0 | 361 | | 2000 |

Table S7. Medicine

| R | Journal Name | TC | TP | H | TC/TP | Year | Volume | IF | IF5 | T50 | T200 | T200* | GR | First Year |
| --- | --- | --- | --- | --- | --- | --- | --- | --- | --- | --- | --- | --- | --- | --- |
| 1 | American Journal of Tropical Medicine and Hygiene | 178865 | 7809 | 120 | 22,9 | 1990 | 42 | 2,736 | 2,947 | 9 | 1 | 1 | 6 | 1921 |
| 2 | Journal of General Internal Medicine | 114351 | 4688 | 120 | 24,39 | 1990 | 5 | 3,423 | 3,744 | 14 | 3 | 3 | 13 | 1986 |
| 3 | Academic Medicine | 92662 | 7000 | 98 | 13,24 | 1990 | 65 | 3,468 | 3,654 | 12 | 1 | 1 | 17 | 1926 |
| 4 | Transactions of The Royal Society of Tropical Medicine and Hygiene | 84422 | 4933 | 83 | 17,11 | 1990 | 84 | 1,931 | 2,453 | 1 | 0 | 0 | 20 | 1908 |
| 5 | Psychiatric Services | 74932 | 4731 | 91 | 15,84 | 1995 | 46 | 1,987 | 2,807 | 6 | 0 | 0 | 24 | 1950 |
| 6 | Journal of Pain and Symptom Management | 71194 | 3586 | 95 | 19,85 | 1991 | 6 | 2,737 | 3,240 | 2 | 0 | 0 | 27 | 1986 |
| 7 | Tropical Medicine & International Health | 56435 | 3196 | 74 | 17,66 | 1996 | 1 | 2,302 | 2,953 | 0 | 0 | 0 | 35 | 1996 |
| 8 | Nicotine & Tobacco Research | 28496 | 1975 | 61 | 14,43 | 2003 | 5 | 2,805 | 3,125 | 2 | 0 | 0 | 69 | 1999 |
| 9 | Palliative Medicine | 25715 | 1711 | 63 | 15,03 | 1995 | 9 | 2,845 | 3,565 | 0 | 0 | 0 | 71 | 1987 |
| 10 | Journal of Womens Health | 20943 | 1932 | 49 | 10,84 | 1997 | 6 | 1,896 | 1,989 | 1 | 0 | 0 | 85 | 1992 |
| 11 | Journal of Manipulative and Physiological Therapeutics | 18094 | 2429 | 45 | 7,45 | 1990 | 13 | 1,248 | 1,471 | 1 | 1 | 1 | 101 | 1978 |
| 12 | Annals of Human Biology | 15402 | 1414 | 44 | 10,89 | 1990 | 17 | 1,148 | 1,515 | 0 | 0 | 0 | 111 | 1974 |
| 13 | Journal of Evaluation in Clinical Practice | 13335 | 1785 | 44 | 7,47 | 1999 | 5 | 1,580 | 1,534 | 0 | 0 | 0 | 118 | 1995 |
| 14 | Vector-Borne and Zoonotic Diseases | 13124 | 1248 | 41 | 10,52 | 2003 | 3 | 2,531 | 2,635 | 0 | 0 | 0 | 124 | 2001 |
| 15 | Journal of Public Health Dentistry | 11983 | 1012 | 46 | 11,84 | 1990 | 50 | 1,644 | 1,653 | 0 | 0 | 0 | 130 | 1941 |
| 16 | Journal of Palliative Medicine | 11142 | 1586 | 39 | 7,03 | 2005 | 8 | 2,063 | 2,446 | 0 | 0 | 0 | 138 | 1987 |
| 17 | European Journal of Cancer Care | 9674 | 1056 | 40 | 9,16 | 1999 | 8 | 1,762 | 1,813 | 0 | 0 | 0 | 147 | 1992 |
| 18 | Journal of Medical Screening | 9179 | 675 | 41 | 13,6 | 1998 | 5 | 2,722 | 2,234 | 0 | 0 | 0 | 150 | 1994 |
| 19 | Implementation Science | 8777 | 883 | 39 | 9,94 | 2006 | 1 | 3,470 | 4,098 | 1 | 0 | 0 | 155 | 2006 |
| 20 | Medical Anthropology Quarterly | 8041 | 608 | 40 | 13,23 | 1990 | 4 | 0,607 | 1,117 | 0 | 0 | 0 | 159 | 1987 |
| 21 | Journal of Palliative Care | 7189 | 727 | 37 | 9,89 | 1995 | 11 | 0,803 | 1,144 | 0 | 0 | 0 | 169 | 1985 |
| 22 | Tropical Doctor | 7162 | 2698 | 22 | 2,65 | 1990 | 20 | 0,528 | 0,576 | 0 | 0 | 0 | 170 | 1971 |
| 23 | Fluoride | 6002 | 790 | 32 | 7,6 | 1990 | 23 | 0,931 | 1,073 | 0 | 0 | 0 | 183 | 1968 |
| 24 | High Altitude Medicine & Biology | 5323 | 584 | 31 | 9,11 | 2001 | 2 | 1,818 | 1,945 | 0 | 0 | 0 | 188 | 2000 |
| 25 | Prehospital Emergency Care | 4415 | 666 | 25 | 6,63 | 2006 | 10 | 1,806 | 1,78 | 0 | 0 | 0 | 201 | 1997 |
| 26 | Bulletin of The History of Medicine | 3438 | 481 | 21 | 7,15 | 1990 | 64 | 0,529 | 0,652 | 1 | 0 | 0 | 214 | 1933 |
| 27 | Medical History | 2512 | 563 | 18 | 4,46 | 1990 | 34 | 0,556 | 0,63 | 0 | 0 | 0 | 229 | 1957 |
| 28 | American Journal of Hospice & Palliative Medicine | 1983 | 629 | 17 | 3,15 | 2008 | 25 | 1,347 | 1,463 | 0 | 0 | 0 | 240 | 1984 |
| 29 | Journal of The History of Medicine and Allied Sciences | 1960 | 412 | 17 | 4,76 | 1990 | 45 | 0,686 | 0,775 | 0 | 0 | 0 | 241 | 1946 |
| 30 | Southeast Asian Journal of Tropical Medicine and Public Health | 1898 | 949 | 13 | 2 | 2009 | 40 | 0,546 | N/A | 0 | 0 | 0 | 242 | 1971 |
| 31 | Asian Pacific Journal of Tropical Medicine | 1822 | 1180 | 13 | 1,54 | 2008 | 1 | 0,926 | 0,665 | 0 | 0 | 0 | 246 | 2008 |
| 32 | Psychology Health & Medicine | 1748 | 403 | 18 | 4,34 | 2009 | 14 | 1,532 | N/A | 0 | 0 | 0 | 250 | 1996 |
| 33 | Travel Medicine and Infectious Disease | 1223 | 366 | 16 | 3,34 | 2009 | 7 | 1,538 | N/A | 0 | 0 | 0 | 264 | 2003 |
| 34 | Annali dell Istituto Superiore di Sanita | 1121 | 361 | 15 | 3,11 | 2008 | 44 | 0,773 | 0,962 | 0 | 0 | 0 | 269 | 1965 |
| 35 | Journal of Pediatric Health Care | 1022 | 278 | 13 | 3,68 | 2008 | 22 | 1,970 | 1,820 | 0 | 0 | 0 | 273 | 1987 |
| 36 | Reproductive Health | 783 | 274 | 13 | 2,86 | 2010 | 7 | 1,616 | N/A | 0 | 0 | 0 | 285 | 2004 |
| 37 | Puerto Rico Health Sciences Journal | 595 | 299 | 10 | 1,99 | 2008 | 27 | 0,667 | 0,687 | 0 | 0 | 0 | 298 | 1982 |
| 38 | Anales del Sistema Sanitario de Navarra | 577 | 495 | 10 | 1,17 | 2007 | 30 | 0,557 | 0,425 | 0 | 0 | 0 | 300 | 1978 |
| 39 | Journal of Developmental Origins of Health and Disease | 557 | 250 | 9 | 2,23 | 2010 | 1 | 0,765 | 0,937 | 0 | 0 | 0 | 303 | 2010 |
| 40 | Journal of Mens Health | 485 | 251 | 10 | 1,93 | 2008 | 5 | 0,678 | 0,746 | 0 | 0 | 0 | 311 | 2004 |
| 41 | Diving and Hyperbaric Medicine | 484 | 274 | 9 | 1,77 | 2007 | 34 | 0,607 | 0,587 | 0 | 0 | 0 | 312 | 1971 |
| 42 | Medycyna Pracy | 416 | 446 | 6 | 0,93 | 2008 | 59 | 0,318 | 0,316 | 0 | 0 | 0 | 316 | 1950 |
| 43 | Journal of Nepal Medical Association | 386 | 402 | 7 | 0,96 | 2008 | 47 | 0,174 | 0,340 | 0 | 0 | 0 | 318 | 1963 |
| 44 | BMC Palliative Care | 327 | 148 | 8 | 2,21 | 2011 | 10 | 1,78 | N/A | 0 | 0 | 0 | 324 | 2002 |
| 45 | Pathogens and Global Health | 324 | 184 | 8 | 1,76 | 2012 | 106 | 0,841 | 0,841 | 0 | 0 | 0 | 327 | 1907 |
| 46 | Medicc Review | 256 | 178 | 6 | 1,44 | 2008 | 10 | 0,582 | 0,816 | 0 | 0 | 0 | 332 | 1999 |
| 47 | Malawi Medical Journal | 205 | 186 | 6 | 1,1 | 2008 | 20 | 0,191 | 0,362 | 0 | 0 | 0 | 341 | 1986 |
| 48 | Tobacco Induced Diseases | 161 | 82 | 7 | 1,96 | 2011 | 9 | 1,500 | N/A | 0 | 0 | 0 | 345 | 2002 |
| 49 | Journal of Arthropod-Borne Diseases | 62 | 73 | 3 | 0,85 | 2012 | 6 | 0,750 | 0,750 | 0 | 0 | 0 | 356 | 2007 |
| 50 | Anthropology & Medicine | 51 | 27 | 4 | 0,93 | 2014 | 21 | N/A | N/A | 0 | 0 | 0 | 363 | 1994 |
| 51 | Journal of The American Association of Nurse Practitioners | 49 | 141 | 3 | 0,35 | 2013 | 25 | N/A | N/A | 0 | 0 | 0 | 358 | 1989 |

Table S8. Health Informatics, Engineering and Technology (e-health)

| R | Journal Name | TC | TP | H | TC/TP | Year | Volume | IF | IF5 | T50 | T200 | T200* | GR | First Year |
| --- | --- | --- | --- | --- | --- | --- | --- | --- | --- | --- | --- | --- | --- | --- |
| 1 | Journal of The American Medical Informatics Association | 47258 | 3139 | 94 | 15,06 | 2000 | 7 | 3,932 | 4,182 | 26 | 0 | 0 | 40 | 1994 |
| 2 | Health Physics | 39417 | 4799 | 62 | 8,21 | 1990 | 58 | 0,774 | 1,105 | 4 | 1 | 1 | 52 | 1958 |
| 3 | International Journal of Medical informatics | 22736 | 1696 | 55 | 13,41 | 1997 | 44 | 2,716 | - | 5 | 0 | 0 | 80 | 1970 |
| 4 | Methods of information in Medicine | 17886 | 1704 | 47 | 10,5 | 1990 | 24 | 1,083 | 1,448 | 3 | 0 | 0 | 102 | 1962 |
| 5 | Journal of Telemedicine and Telecare | 16334 | 1980 | 45 | 8,25 | 1998 | 4 | 1,736 | 1,661 | 0 | 0 | 0 | 106 | 1995 |
| 6 | Journal of Medical Internet Research | 15683 | 1271 | 53 | 12,34 | 1999 | 1 | 4,669 | 5,724 | 4 | 0 | 0 | 108 | 1999 |
| 7 | International Journal of Technology Assessment in Health Care | 15141 | 1346 | 45 | 11,25 | 1995 | 11 | 1,556 | 1,565 | 3 | 0 | 0 | 112 | 1985 |
| 8 | Health Technology Assessment | 13181 | 650 | 56 | 20,28 | 2004 | 8 | 5,116 | 5,404 | 5 | 1 | 0 | 123 | 1997 |
| 9 | Journal of Medical Systems | 5709 | 1188 | 26 | 4,81 | 1992 | 16 | 1,372 | 1,482 | 0 | 0 | 0 | 186 | 1977 |
| 10 | International Journal of Health Geographics | 4350 | 485 | 28 | 8,97 | 2007 | 6 | 1,967 | 2,675 | 0 | 0 | 0 | 202 | 2002 |
| 11 | Journal of Radiological Protection | 3088 | 646 | 23 | 4,78 | 2003 | 23 | 1,319 | 1,488 | 0 | 0 | 0 | 220 | 1981 |
| 12 | Geospatial Health | 1835 | 293 | 20 | 6,26 | 2006 | 1 | 1,000 | 1,479 | 0 | 0 | 0 | 245 | 2006 |
| 13 | Telemedicine and e-Health | 1503 | 569 | 15 | 2,64 | 2011 | 17 | 1,544 | 1,753 | 0 | 0 | 0 | 254 | 1995 |
| 14 | Informatics for Health & Social Care | 535 | 140 | 12 | 3,82 | 2008 | 33 | 0,711 | 0,979 | 0 | 0 | 0 | 305 | 1976 |
| 15 | Health Informatics Journal | 498 | 141 | 11 | 3,53 | 2009 | 15 | 0,787 | N/A | 0 | 0 | 0 | 309 | 1995 |
| 16 | Technology and Health Care | 416 | 287 | 9 | 1,45 | 1993 | 1 | 0,636 | N/A | 0 | 0 | 0 | 317 | 1993 |
| 17 | Journal of Healthcare Engineering | 248 | 152 | 7 | 1,63 | 2010 | 1 | 0,468 | 0,598 | 0 | 0 | 0 | 333 | 2010 |
| 18 | Simulation in Healthcare-Journal of The Society for Simulation in Healthcare | 167 | 137 | 6 | 1,22 | 2008 | 3 | 1,593 | 1,960 | 0 | 0 | 0 | 343 | 2006 |

Table S9. Primary Health Care

| R | Journal Name | TC | TP | H | TC/TP | Year | Volume | IF | IF5 | T50 | T200 | T200* | GR | First Year |
| --- | --- | --- | --- | --- | --- | --- | --- | --- | --- | --- | --- | --- | --- | --- |
| 1 | British Journal of General Practice | 47635 | 5770 | 74 | 8,26 | 1990 | 40 | 2,356 | 2,516 | 8 | 0 | 0 | 39 | 1953 |
| 2 | American Family Physician | 43288 | 5726 | 62 | 7,56 | 1990 | 41 | 1,818 | 2,056 | 4 | 0 | 0 | 47 | 1970 |
| 3 | Journal of Family Practice | 37511 | 3371 | 78 | 11,13 | 1990 | 30 | 0,735 | 0,729 | 17 | 1 | 1 | 55 | 1974 |
| 4 | Family Practice | 35533 | 2322 | 66 | 15,3 | 1990 | 7 | 1,842 | 2,071 | 6 | 0 | 0 | 58 | 1984 |
| 5 | Annals of Family Medicine | 16159 | 666 | 59 | 24,26 | 2004 | 2 | 4,570 | 5,250 | 9 | 0 | 0 | 107 | 2003 |
| 6 | Canadian Family Physician | 13319 | 5046 | 35 | 2,64 | 1990 | 36 | 1,403 | 1,646 | 0 | 0 | 0 | 120 | 1955 |
| 7 | Family Medicine | 10804 | 1546 | 38 | 6,99 | 2000 | 32 | 0,851 | 1,284 | 3 | 1 | 1 | 139 | 1981 |
| 8 | Scandinavian Journal of Primary Health Care | 9132 | 866 | 35 | 10,55 | 1995 | 13 | 1,610 | 1,889 | 0 | 0 | 0 | 152 | 1983 |
| 9 | Journal of The American Board of Family Medicine | 7230 | 898 | 32 | 8,05 | 2006 | 19 | 1,848 | 2,064 | 2 | 0 | 0 | 168 | 1988 |
| 10 | Primary Care | 6856 | 1244 | 28 | 5,51 | 1990 | 17 | 0,833 | 1,084 | 1 | 0 | 0 | 174 | 1974 |
| 11 | Physician and Sportsmedicine | 5734 | 2391 | 25 | 2,4 | 1990 | 18 | 1,490 | 1,308 | 0 | 0 | 0 | 185 | 1973 |
| 12 | BMC Family Practice | 4806 | 993 | 25 | 4,84 | 2007 | 8 | 1,735 | 2,144 | 0 | 0 | 0 | 196 | 2000 |
| 13 | Australian Family Physician | 3340 | 1538 | 18 | 2,17 | 2007 | 36 | 0,668 | 0,712 | 0 | 0 | 0 | 216 | 1972 |
| 14 | Atencion Primaria | 1020 | 1064 | 10 | 0,96 | 2007 | 39 | 0,894 | 0,722 | 0 | 0 | 0 | 274 | 1984 |
| 15 | Australian Journal of Primary Health | 907 | 385 | 11 | 2,36 | 2007 | 13 | 1,219 | 1,039 | 0 | 0 | 0 | 281 | 1995 |
| 16 | Primary Care Diabetes | 559 | 196 | 10 | 2,85 | 2010 | 4 | 1,289 | N/A | 0 | 0 | 0 | 302 | 2007 |
| 17 | European Journal of General Practice | 496 | 195 | 10 | 2,54 | 2004 | 10 | 0,810 | N/A | 0 | 0 | 0 | 310 | 1995 |
| 18 | NPJ Primary Care Respiratory Medicine | 17 | 52 | 2 | 0,33 | 2014 | 24 | 2,909 | N/A | 0 | 0 | 0 | 360 | 1991 |
